# Fully-automated sleep staging for Parkinson’s disease and isolated REM sleep behavior disorder

**DOI:** 10.1038/s41746-026-02946-2

**Published:** 2026-08-11

**Authors:** Jesper Strøm, Casper Skjærbæk, Natasha Becker Bertelsen, Steffen Torpe Simonsen, Niels Okkels, David Bertram, Sinah Röttgen, Konstantin Kufer, Kaare B. Mikkelsen, Marit Otto, Poul Jørgen Jennum, Per Borghammer, Michael Sommerauer, Preben Kidmose

**Affiliations:** 1 Department of Electrical and Computer Engineering, Aarhus University, Aarhus, Denmark; 2 Department of Nuclear Medicine, Aarhus University Hospital, Aarhus, Denmark; 3 Lundbeck Foundation Parkinson’s Disease Research Center (PACE), Aarhus University, Aarhus, Denmark; 4 Department of Neurology, Aarhus University Hospital, Aarhus, Denmark; 5 Department of Clinical Medicine, Aarhus University, Aarhus, Denmark; 6 Faculty of Mathematics and Natural Sciences, University of Cologne, Cologne, Germany; 7 Institute for Biomedical Informatics, Faculty of Medicine and University Hospital Cologne, Cologne, Germany; 8 Center for Molecular Medicine Cologne (CMMC), Faculty of Medicine and University Hospital Cologne, Cologne, Germany; 9 Cognitive Neuroscience, Institute for Neuroscience and Medicine, INM-3, Research Center Juelich, Juelich, Germany; 10 Department of Neurology, Faculty of Medicine and University Hospital Cologne, University of Cologne, Cologne, Germany; 11 Center of Neurology, Department of Parkinson, Sleep and Movement Disorders, University Hospital Bonn, University of Bonn, Bonn, Germany; 12 German Center for Neurodegenerative Diseases (DZNE), Bonn, Germany; 13 Danish Center for Sleep Medicine, Rigshospitalet, Glostrup, Denmark

**Keywords:** Diseases, Medical research, Neurology, Neuroscience

## Abstract

Isolated REM sleep behavior disorder (iRBD) is a key prodromal marker of Parkinson’s disease (PD). Video-polysomnography (vPSG) remains the diagnostic gold standard, but manual sleep staging is particularly time-consuming and challenging in neurodegenerative disease. We adapted U-Sleep, a deep neural network, for automated sleep staging in PD and iRBD. A pretrained model (PUB, 19,236 PSGs), was finetuned on multicenter datasets (PACE, CBC: 112 PD, 138 iRBD, 89 controls) and evaluated on a clinical hold-out (DCSM: 81 PD, 36 iRBD, 87 controls). Predictors of staging agreement were analyzed, and low-agreement recordings were blindly rescored. Confidence-based thresholds were applied to enhance REM detection. The pretrained model achieved *κ* = 0.66 in PACE/CBC, improving to *κ* = 0.74 after finetuning (*p* < 0.001). In the hold-out, mean κ increased from 0.60 to 0.64 (*p* < 0.001). Site-specific finetuning provided minimal benefit. Confidence was a significant predictor of Cohen’s κ (*p* < 0.001). Recordings with low model agreement also showed low human interrater agreement. Applying a confidence threshold increased REM precision from 85 to 95.6%, preserving sufficient REM sleep in 96% of subjects. This publicly available model achieves human-level agreement enabling scalable, standardized PSG analysis with model-derived confidence as a tool for further refinements.

## Introduction

Over the past 25 years, the global burden of Parkinson’s disease (PD) has more than doubled, and PD is now the fastest-growing neurological disorder in terms of prevalence, disability, and deaths^1^, with further increases anticipated through 2050^2^. PD belongs to the group of α-synucleinopathies together with dementia with Lewy bodies (DLB), multiple system atrophy (MSA), and isolated REM sleep behavior disorder (iRBD). RBD is characterized by dream-enactment behaviors, such as shouting, kicking, and complex movements during vivid dreaming in REM sleep. Although these symptoms may appear benign, iRBD, in most cases, reflects an underlying α-synucleinopathy affecting the brainstem^3^. As the strongest prodromal marker of PD, the likelihood of later PD is increased by approximately 130-fold^4^. After 12–14 years of follow-up, 73–90% of individuals with iRBD develop an overt α-synucleinopathy^5,6^. As a result, iRBD is fast becoming a key entry point for α-synucleinopathy trials^7^.

Extensive screening efforts seek to identify iRBD before the onset of overt motor or cognitive symptoms. These include community-based questionnaire screening^8–10^, device-based approaches using actigraphy and wearable EEG/EMG^11–14^, and, more recently, assays of α-synuclein aggregation in blood or cerebrospinal fluid^15,16^. But, none currently provides sufficient sensitivity and specificity to replace video-polysomnography (vPSG) as the diagnostic gold standard for REM sleep behavior disorder (RBD). Guideline-based evaluation relies on at least one vPSG, typically performed in-hospital, to document REM sleep without atonia (RSWA) and dream enactment behaviors^17^, and requires multichannel EEG, electrooculography (EOG), multiple electromyography (EMG) channels, cardiorespiratory monitoring, and synchronized video recording. Sleep stages are then manually assigned to consecutive 30-s epochs (≈1000 per night) in accordance with the AASM Scoring Manual^18^. As questionnaire- and biomarker-based screening scales up, labor-intensive confirmatory vPSG assessments become a bottleneck to the deployment of new technologies for RBD screening.

Sleep staging in neurodegenerative disease requires substantial expertise at each sleep center to provide consistent results. In healthy adults, interrater agreement when applying AASM rules is substantial, with Cohen’s *κ* values around 0.76^19^. In PD and related disorders, pathological changes in sleep EEG are common, including EEG slowing during wakefulness^20,21^, attenuation or loss of N2 hallmarks such as sleep spindles^22,23^, and reduced or atypical rapid eye movements during REM sleep^24^. These deviations from canonical sleep architecture undermine the standard AASM rules and reduce interrater agreement, with Cohen’s *κ* ≈ 0.61 reported in the largest PD study^25^ and slightly higher values in a smaller study^26^. In neurodegenerative disease, strict application of AASM rules may, in some cases, be impractical or impossible, for example, in patients without definite rapid eye movements or with absence of REM atonia. To address this, RBD-specific staging recommendations for REM sleep and modified criteria for sleep in neurodegenerative disease have been proposed^17,23,27^. While such adaptations may improve consistency within a center, they also increase complexity and introduce site-specific practices and inconsistent staging, which complicate the analysis of human-scored data and limit the performance of supervised models trained on these annotations.

Automated sleep staging could bypass such variability and alleviate the current bottleneck of manual sleep staging. Deep neural network models have advanced substantially in recent years, now achieving agreement with human raters comparable to human–human interrater agreement in non-neurodegenerative datasets^28,29^. U-Sleep, a fully convolutional neural network, robustly reproduces staging across diverse non-neurodegenerative populations when trained on large multisite datasets^28,30^. Yet models are typically trained on datasets dominated by healthy adults, and none have been adapted or validated for use in neurodegenerative diseases. Domain shift due to pathological EEG, fragmented sleep, comorbid sleep disorders, and inconsistent human labels may substantially degrade performance. This is supported by prior work applying a random-forest classifier to PSG from individuals with iRBD, in which agreement was substantial in elderly controls (*κ* = 0.73) but dropped markedly in iRBD (*κ* = 0.54), with less than half of manually scored REM sleep correctly identified^31^. This pattern underscores that models performing well in healthy cohorts may fail in challenging populations, including iRBD.

A prerequisite for scalable use of PSG in neurodegenerative disease is a robust, generalizable sleep-staging model that maintains near-human agreement across centers, disease stages, and comorbid sleep disorders, and that provides calibrated measures of confidence to guide manual review. Our objective was to adapt and validate the U-Sleep model for application in PD, iRBD, and clinically relevant controls across multiple sites. We fine-tuned a pretrained U-Sleep model, initially trained on large, multisite, non-neurodegenerative datasets, using high-quality research PSGs from two research centers, comprising individuals with iRBD, PD without co-morbid RBD (PD^−RBD^), PD with RBD (PD^+RBD^), and age-matched controls recruited primarily through questionnaire-based screening initiatives^8,9^ and a sleep clinic. Importantly, we did not exclude co-morbid sleep disorders such as OSA and insomnia, to reflect populations encountered in clinical and screening settings.

To inform clinical deployment, we then addressed five key questions: (i) how much performance degrades when the pretrained model is applied directly to neurodegenerative datasets and to what extent finetuning restores agreement, including whether a single generalized model can replace site-specific models; (ii) how well the generalized model performs in an independent hold-out cohort; (iii) which factors (e.g., age, apnea–hypopnea index, U-Sleep confidence) determine agreement; (iv) whether, in challenging recordings, a blinded second human scorer agrees more closely with the model or the original scorer, thereby revealing signs of overfitting or systematic deviations (v) whether model confidence can be used to develop purpose-specific adaptations, most importantly, optimized REM sleep detection using thresholds preserving sufficient REM sleep duration for downstream clinical review or automated RSWA assessment in PD and iRBD.

## Results

We first replicated the original U-Sleep model on a large, publicly available multisite dataset of healthy individuals, children, and patients with obstructive sleep apnea (PUB; 19,236 PSG recordings across 12 sites; Table S1), achieving a validation Cohen’s *κ* of 0.813 and a per-night *κ* of 0.818 on the test set. This *Pretrained Model* was then fine-tuned on two high-quality research datasets (PACE and CBC) comprising patients with neurodegenerative disorders (112 PD and 138 iRBD) and age-matched controls (*n* = 89; Table 4), resulting in a *Generalized Model*. Additionally, *Site-Specific Models* were trained on PACE and CBC individually. Finally, the Generalized Model was evaluated on an independent clinical hold-out dataset from the Danish Centre of Sleep Medicine (DCSM), enriched with patients with neurodegenerative disorders (81 PD and 36 iRBD) and sleep-clinic patients without neurodegenerative disease (*n* = 87). The Generalized Model is available on our GitHub repository (https://github.com/jesperstroem/U-Sleep-for-RBD-PD).

### Sleep staging performance

A total of 19,236 PSG recordings were included in PUB (Table S1) for the multicenter pretrained model. Per-night *κ* and stage-wise F1-scores for the reproduced Pretrained Model are presented in Table S2. As we used a different data stratification and computed κ and F1 on a per-night rather than a per-dataset basis, these scores are not directly comparable to those reported by Perslev et al.^28^. To demonstrate comparable performance, per-dataset F1-scores for both models are reported in Table S3.

**Fig. 1:**
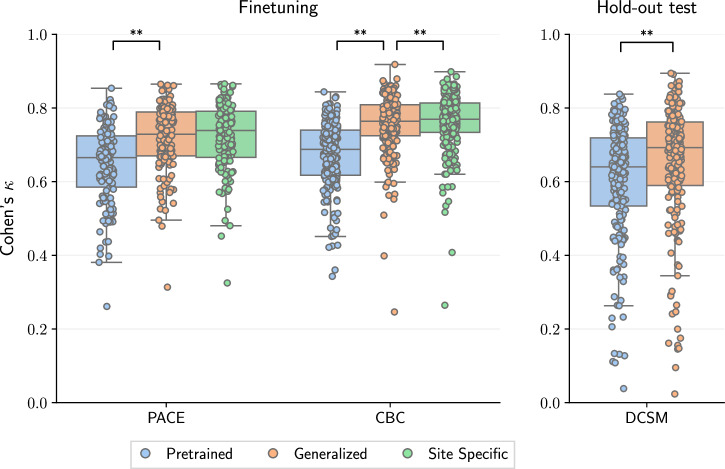
Distribution of per-night *κ* values for each model when applied to datasets individually. Left panel: The Pretrained Model, similar to the model in Perslev et al., achieved median *κ* values of 0.67 and 0.69 for PACE and CBC, respectively, and improved significantly upon fine-tuning. Only in CBC, is the Site-Specific Model marginally better than the Generalized Model. Right panel: The DCSM dataset was used as the hold-out set, and median *κ* increased from 0.64 to 0.69 using the Generalized Model compared to the Pretrained Model. *Indicates significant improvements (*p < 0.05, **p < 0.001).

Fine-tuned models were developed for the neurodegenerative datasets. The distribution of per-night κ scores between the model and the human scorer is shown in Fig. 1 for all three model types: the Pretrained Model, the Generalized Model fine-tuned on the PACE and CBC datasets with neurodegenerative patients, and the Site-Specific Models trained on a single center, all evaluated using 10-fold cross-validation. Accuracy, *κ*, and F1-scores for each model are provided in Table 1 with confusion matrices provided in Fig. S1 and the metrics for each dataset provided in Table S5.

**Table 1:** Comparison of model performance and sleep architecture across evaluated models

	Metric	Human	Pretrained	Generalized	Site Specific
	Accuracy		0.76 ± 0.07	0.82 ± 0.06	0.82 ± 0.06
	Cohen’s κ		0.66 ±0.10	0.74 ± 0.09	0.75 ± 0.09
**F1 Scores**	W		0.74 ± 0.13	0.82 ± 0.11	0.82 ± 0.11
	N1		0.39 ± 0.14	0.52 ± 0.15	0.53 ± 0.16
	N2		0.81 ± 0.08	0.84 ± 0.07	0.84 ± 0.07
	N3		0.74 ± 0.17	0.80 ± 0.14	0.80 ± 0.14
	R		0.80 ± 0.16	0.87 ± 0.11	0.87 ± 0.11
	Average		0.70 ± 0.07	0.77 ± 0.07	0.77 ± 0.07
**Sleep architecture**	Total Sleep Time, TST (m)	396.27 ± 67.89	401.76 ± 64.24**	396.64 ± 64.63	396.61 ± 63.51
	Sleep Efficiency, SEFF (%)	80.7 ± 10.84	81.83 ± 9.88*	80.78 ± 10.08	80.83 ± 10.23
	Sleep Onset Latency, SOL (m)	11.15 ± 12.91	12.08 ± 14.73	12.41 ± 13.94	12.57 ± 14.66
	REM Latency, REMLAT (m)	124.44 ± 81.11	124.37 ± 84.99**	118.05 ± 76.22**	116.6 ± 74.79**
	Fraction of N1, PN1 (%)	16.2 ± 6.67	16.14 ± 7.31**	17.37 ± 6.99**	17.17 ± 6.91**
	Fraction of N2, PN2 (%)	17.81 ± 11.31	12.27 ± 8.86**	14.38 ± 8.91**	14.36 ± 9.75*
	Fraction of N3, PN3 (%)	50.25 ± 10.23	57.33 ± 10.13**	51.69 ± 9.57*	51.91 ± 10.2*
	Fraction of R, PREM (%)	15.75 ± 7.62	14.26 ± 8.05	16.56 ± 7.45**	16.55 ± 7.45**
	Wake After Sleep Onset, WASO (m)	80.55 ± 55.02	79.16 ± 50.77	83.94 ± 52.29**	83.82 ± 52.91
	Stage Changes (n)	145.87 ± 53.99	108.41 ± 41.06**	128.09 ± 47.83**	129.22 ± 48.85**

Mean and standard deviation of *κ*, F1 scores, and sleep architecture measures for each model evaluated on PACE and CBC. Stars indicate a significant difference from human scorings (**p < 0.001, *p < 0.05).

Across 339 research recordings in total (PACE = 134, CBC = 205, Table S4), average per-night κ increased significantly from the Pretrained Model to the Generalized Model at both sites (*p* < 0.001), and F1-scores increased across all sleep stages (*p* < 0.001). Site-Specific Models yielded a statistically significant improvement only for CBC (PACE: *p* = 0.076; CBC: *p* < 0.001). When combining stages N1 and N2 into *Light Sleep*, the overall *κ* of all models increased significantly (*p* < 0.001), and for the Generalized Model, the F1-score for Light Sleep reached 0.88 (Table S9).

For the unseen “hold-out” DCSM dataset (Fig. 1, right panel), the Pretrained Model without finetuning produced a mean per-night *κ* of 0.60, which improved significantly to 0.64 with the Generalized Model (*p* < 0.001). Median per-night *κ* also improved from *κ* = 0.64 to *κ* = 0.69 (*p* < 0.001). Although neither the Pretrained nor the Generalized Model was trained on DCSM data, finetuning specifically to the DCSM site did not yield significant improvements in median κ or stage-wise F1 scores compared to using the Generalized Model (*κ* = 0.69 vs. 0.70, *p* = 0.42).

Finetuning the Pretrained Model resulted in significant improvements across all sleep stages compared to the Pretrained Model on PACE and CBC (*p* < 0.001 for all sleep stages). Site-Specific Models have significantly better staging of Wake, N1 and REM (W: *p* = 0.013, N1: *p* = 0.009, N2: *p* = 0.838, N3: *p* = 0.467, R: *p* = 0.005).

The datasets comprise four clinical subgroups, including PD patients without RBD (PD^-RBD^), PD patients with RBD (PD^+RBD^), iRBD, and controls, as shown in Table 4. Controls in PACE were slightly older than patients, although not significant (*p* = 0.06), while controls in CBC were significantly younger than patients (*p* = 0.01). For both PACE and CBC, controls tended to have higher apnea-hypopnea index, although only significant for CBC (PACE: *p* = 0.10, CBC: *p* < 0.001, respectively). In contrast, controls from DCSM were younger and had lower AHI than patients (*p* < 0.001, *p* = 0.011, respectively).

**Fig. 2:**
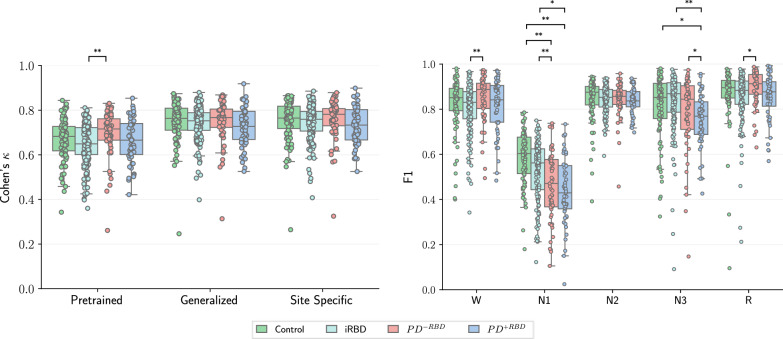
Performance on clinical subgroups. Left: Distribution of per-night *κ* scores for the models across clinical subgroups in PACE and CBC. A significant difference was found between iRBD and PD^-RBD^ for the Pretrained Model. For comparison, a meta-analysis of interrater agreement in healthy controls found a *κ* = 0.76, and a study of interrater agreement in patients with PD found a *κ* = 0.61^19,25^. Right: Stage-wise performance across each clinical subgroup in PACE and CBC for the Generalized Model. * Indicates significant improvements (* *p* < 0.05, ** *p* < 0.001).

For PACE and CBC, no significant differences in median per-night *κ* were observed across clinical subgroups, except for the Pretrained Model on iRBD and PD^-RBD^ (*p* < 0.001), as shown in Fig. 2 (left). A non-significant difference was observed for the Site-Specific Model between PD^-RBD^ and PD^+RBD^. As shown in Fig. 2 (right) the Generalized Model showed significant differences between groups when comparing stage-wise F1-scores for N1 and N3 in PD patients, with PD^+RBD^ generally having the lowest F1 score (*p* < 0.001). No stage-wise differences were found for W, N2 and R, except in W between iRBD and PD^-RBD^ (*p* < 0.001) and in R between iRBD and PD^-RBD^ (*p* = 0.048). Corresponding means and standard deviations are presented in Table S6, and confusion matrices are shown in Fig. S2. A non-significant positive association was observed between Cohen’s *κ* and disease duration in the PD^-RBD^ and PD^+RBD^ subgroups from PACE and CBC (R^2^ = 0.006, *p* = 0.22, shown in Fig. S3).

In DCSM, the Generalized Model showed significant differences in median κ between controls and all subgroups (Control: *κ* = 0.75, iRBD: *κ* = 0.65, PD^-RBD^: *κ* = 0.59, PD^+RBD^: *κ* = 0.64, *p* < 0.001). Applying a Site-Specific Model for DCSM showed only non-significant improvements within subgroups compared to the Generalized Model (Control: *p* = 0.45, iRBD: *p* = 0.84, PD^-RBD^: *p* = 0.12, PD^+RBD^: *p* = 1.0).

Sleep architecture measures for PACE and CBC are shown in Table 1 and Fig. S3. For the Pretrained Model, median values differed significantly from human scoring across all parameters (*p* = 0.001 for SEFF; *p* < 0.001 for the other parameters), except for SOL (*p* = 1.0), WASO (*p* = 1.0), and PREM (*p* = 0.16). The Generalized and Site-Specific Models also differ on most parameters, most notably on the number of stage changes, which is markedly lower across all U-sleep models (*p* < 0.001).

### Latency and length of REM sleep and N2 periods

**Fig. 3:**
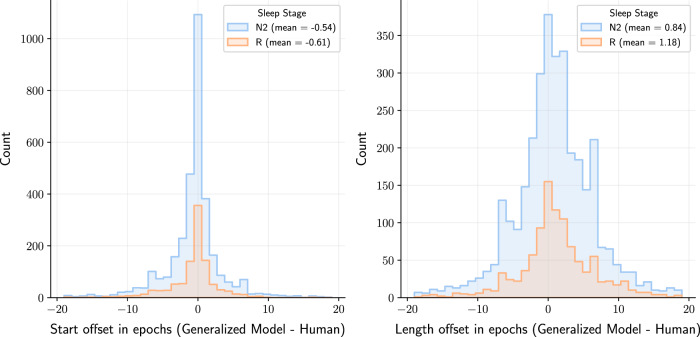
Distributions of start and length offsets for the identified REM sleep and N2 periods. N2 and R periods begin slightly earlier in the sleep stages predicted by the Generalized Model than when staged by human raters, and they continue for longer. This supports the notion that the model does not rely solely on rapid eye movements to initiate REM sleep but has adapted to accommodate the rules for “staging back” REM sleep in contiguous epochs preceding definite R epochs.

In the assessment of REM sleep and N2 period latencies, 1017 overlapping R-periods and 3271 overlapping N2-periods were identified between human labels and the Generalized Model’s sleep staging across all recordings in PACE and CBC. The mean offset was −0.61 epochs for R and −0.54 epochs for N2, indicating that the model, on average, starts classifying both stages earlier than human scorers (*p* < 0.001). The difference in length was 1.18 epochs for R and 0.84 epochs for N2, indicating that the model scores longer consecutive periods of both stages than human raters (*p* < 0.001), as shown in Fig. 3.

### Explaining sleep staging performance

**Fig. 4:**
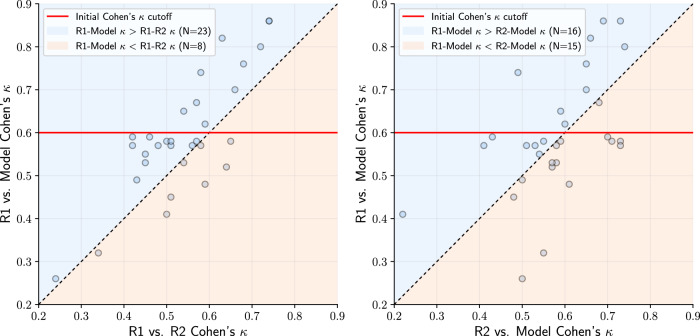
Scatterplots of κ values in the interrater study. The red line indicates the 0.6 *κ* cutoff for rescoring records. The numbers in the left and right panels indicate the number of data points within the areas defined by red and blue colors. Left: Rater 1 (R1) vs. model and Rater 1 (R1) vs. Rater 2 (R2). Overall, better *κ* values were achieved for R1-R2 on only 8/31 PSG recordings, as shown in the lower-right area, suggesting that the agreement between R1 and the model is on par with or exceeds the human interrater agreement. Right: R1 vs. Model and R2 vs. Model. When the initial *κ* between R1 and the model is low, the κ between R2 and the model is often higher, and vice versa. This suggests a “regression towards the mean” effect.

Thirty-one PSG recordings were included in a post hoc interrater study involving a new scorer, blinded to the U-Sleep predictions and the original hypnogram. Of these, 21 recordings were selected because *κ* between the Site-Specific Model and the original scoring was <0.6, and 10 additional recordings with *κ* > 0.6 were selected. The original scorer (Rater 1, R1) and U-Sleep (Model) had a mean *κ* of 0.59, while R1 and the blinded scorer (Rater 2, R2) had a mean *κ* of 0.54. Eight of the chosen PSG recordings had a higher *κ* between R1 and R2 than between R1 and the model, as shown in the left panel of Fig. 4. Mean *κ* between R2 and the model was 0.58, indicating that the model agreed at least as well with each rater as they did with each other.

Table 2 summarizes accuracy, *κ*, and F1 scores for all rater pairs. Consensus *κ* was defined as the *κ* between the model scoring and the epochs in which R1 and R2 agree and reached a mean of 0.74 across all recordings, with *κ* = 0.69 and *κ* = 0.84 for recordings with initial *κ* below or above 0.6, respectively. Across all recordings, R1 and the Model showed higher agreement on N3 (*p* < 0.001) than R1 and R2. For those with low initial *κ*, R1 and R2 showed higher agreement than R2 and the Model only for N1, while R2 and the model reached greater agreement on W than either R1 vs. Model or R1 vs. R2 (*p* < 0.001). When initial *κ* was high, R1 and the model showed significantly higher agreement on average and for N1 and N3 than either R1 vs. R2 or R2 vs. Model (*p* < 0.001). Across all 31 recordings, R1 and R2 show the lowest agreement, as measured by *κ*, but this difference is significant only for R1 vs. Model (*p* = 0.002)

**Table 2:** Comparison of agreement metrics across all rater relations

					F1					
Type	Relation	Accuracy	Cohen’s κ	Human Consensus Cohen’s κ	Wake	N1	N2	N3	R	Average
Initial κ < 0.6	R1 vs. Model	0.67±0.06	0.52±0.09		0.66±0.16	0.38±0.17*	0.72±0.11	0.58±0.27*	0.71±0.26	0.61±0.10
	R1 vs. R2	0.66±0.07	0.49±0.09	0.69±0.13	0.65±0.14	0.47±0.15	0.69±0.15	0.36±0.26	0.70±0.24	0.58±0.07
	R2 vs. Model	0.69±0.10	0.55±0.11		0.77±0.11*	0.45±0.15	0.70±0.18	0.39±0.27	0.70±0.27	0.61±0.10
Initial κ > 0.6	R1 vs. Model	0.82±0.06*	0.75±0.08*		0.83±0.11	0.51±0.12	0.86±0.03*	0.81±0.08*	0.84±0.08	0.77±0.06*
	R1 vs. R2	0.75±0.05	0.64±0.07	0.84±0.06	0.80±0.10	0.49±0.09	0.78±0.06	0.52±0.24	0.83±0.13	0.69±0.07
	R2 vs. Model	0.75±0.05	0.65±0.07		0.82±0.11	0.48±0.11	0.78±0.07	0.54±0.26	0.81±0.15	0.69±0.07

Mean and standard deviation for accuracy, *κ*, and F1 scores for Rater 1 (R1), Rater 2 (R2), and the model. The table is divided in two parts: recordings with initial *κ* < 0.6 in the lower section. Values in bold accompanied by *indicate a statistically significant difference from the two other rater relations within the recording type (*p* < 0.05).

Linear mixed-effects models were used to explore variables associated with per-night *κ* for the Generalized Model on PACE and CBC. Model A included only subject-level information available before clinical PSG, while model B included information available after routine PSG evaluation. Model C included only variables available before PSG or obtained via automatic sleep staging with U-Sleep. The results are shown in Table 3. Model A and B identified age as a significant predictor of κ, and model B also had AHI as a significant predictor with a negative association. This association is illustrated in Fig. S5, comparing per-night *κ*-values for subjects with an AHI below or above 15. Subjects with an AHI above 15 (moderate-severe OSA) had a mean *κ* of 0.71, while subjects with an AHI below 15 (no or mild OSA) had a mean *κ* of 0.75. For model C, U-Sleep confidence was the only statistically significant predictor, with κ expected to increase by 0.011 for each 1%-point increase in confidence. The effect of age remained, with a decrease of 0.001 *κ* per year, although non-significant. The impact of each variable on overall confidence is illustrated in Fig. S5. In 10-fold cross-validation, Model C yielded a mean absolute error of 0.052 in *κ* on PACE and CBC, and the residuals are shown in Fig. S6.

**Table 3:** Overview of regression model parameters, their z scores, and p-values

	Model A: Demographics			Model B: Demographics + Sleep Disorders			Model C: Demographics + U-Sleep		
Parameter	Coef.	z	p	Coef.	z	p	Coef.	z	p
Intercept	0.976	18.853	<0.001*	0.912	17.827	<0.001*	−0.100	−0.918	0.359
Age	−0.003	−5.498	<0.001*	−0.002	−3.827	<0.001*	−0.001	−1.818	0.069
Sex (M)	−0.014	−1.279	0.201	−0.013	−1.199	0.230	0.010	1.078	0.281
BMI	−0.002	−1.290	0.197	−0.000	−0.143	0.887	−0.000	−0.330	0.741
PD	0.009	0.772	0.440	0.005	0.478	0.632	−0.001	−0.076	0.940
AHI				−0.002	−4.329	<0.001*			
RBD				−0.015	−1.588	0.112			
PN1 %							−0.001	−1.008	0.313
STAGEC #							−0.000	−0.232	0.817
U-Sleep Confidence %							0.011	10.836	<0.001*

Model parameters and regression coefficients for the linear mixed-effects models of Cohen’s κ. A missing value indicates that the parameter was not included in the model. PN1% is the percentage of N1 sleep, STAGEC # is the total number of stage changes in the hypnogram, and U–Sleep Confidence % is the percentage of mean confidence of the U–Sleep model when predicting a given sleep stage. * Indicates that a variable has a statistically significant effect on Cohen’s *k* (*p* < 0.001).

**Fig. 5:**
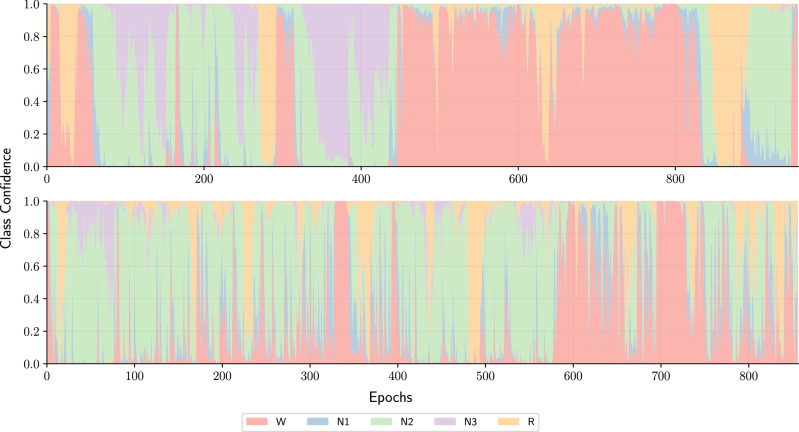
Hypnodensity plots using confidence estimates. Examples of hypnodensity plots with high confidence (top, mean confidence = 0.8785) and low confidence (bottom, mean confidence = 0.6415). For each epoch, the stage-wise confidence sums to 1. In high-confidence cases, each epoch will have a dominant sleep stage with confidence close to 1. In cases with low confidence, the epoch-wise total confidence of 1 will be distributed more evenly between two or more sleep stages. The final sleep stage is the one with the highest confidence, and as more than two sleep stages may have confidence estimates above 0, the final stage’s confidence may be below 0.5 if the model identifies features of multiple sleep stages within the same epoch.

**Fig. 6:**
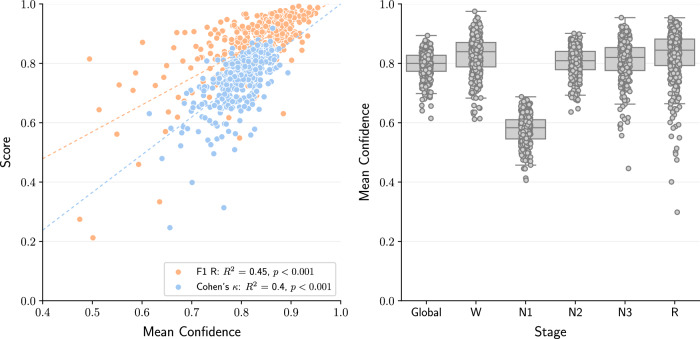
Relationships between model confidence and performance. Left**:** Relationship between overall per-night confidence and per-night *κ* (blue), and the relationship between per-night REM stage confidence and REM F1 score (orange). There is a significant positive association between the model’s per-night confidence and the agreement between its predictions and human scoring. This relationship indicates that confidence estimates can predict agreement with human scoring, as measured by both Cohen’s *κ* and REM F1. Right: Distribution of mean confidence and stage-wise confidence for the PACE and CBC datasets based on the Pretrained and the Generalized Model.

Correlation between the model confidence and *κ* was explored. Epoch-wise distributions of model confidence are exemplified using hypodensity plots in Fig. 5 for a recording with high average confidence (top panel) and low average confidence (bottom panel). For the Generalized Model, the mean confidence across all recordings was 0.80, with W having the highest confidence and N1 the lowest (W = 0.83, N1 = 0.58), as shown in Fig. 6, right. A linear relationship between per-night overall confidence and κ-value was observed (R^2^ = 0.40, *p* < 0.001, Fig. 6, left), and a linear relationship between R-stage confidence and R F1 was also found (R2 = 0.45, *p* < 0.001).

To assess the influence of arousals on model confidence, a total of 13,826 respiratory arousals and 32,426 non-respiratory arousals were manually annotated in 339 PSG recordings in the combined PACE and CBC datasets. The per-night stage-wise distributions of confidence estimates are shown in Fig. S6. Only nights with at least three affected and three unaffected sleep epochs were included. Epochs manually annotated as containing arousals showed significantly lower mean confidence across all sleep stages compared with unaffected epochs, including respiratory arousals, non-respiratory arousals and all arousals combined (W, N1, N2, N3, R: *p* < 0.001). The mean confidence values stratified by sleep stage and arousal type are provided in Table S7.

### REM sleep staging using confidence-based thresholding

**Fig. 7:**
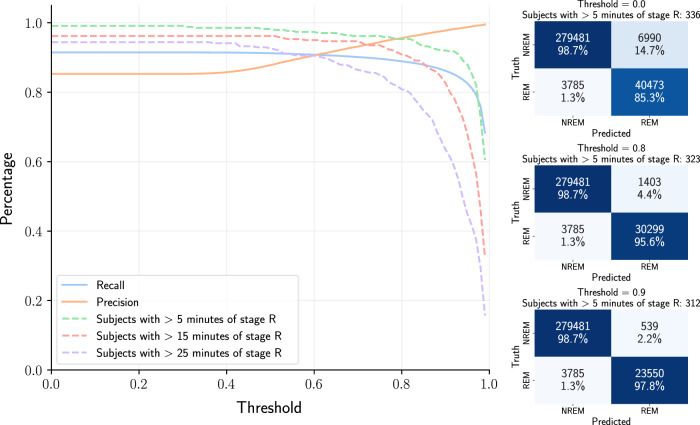
Using confidence thresholds to optimize staging of REM sleep. Left: Stage R recall and precision as a function of U-sleep confidence threshold. The dashed curves indicate the proportion of participants retaining at least 10, 30, or 50 REM sleep epochs at each threshold. Five minutes (10 epochs) of REM sleep is considered the minimum duration of REM sleep needed for subsequent analysis of RSWA. Right: Stage R confusion matrices for arbitrary thresholds that may be implemented to optimize subsequent clinical or automated analyses. Applying a confidence threshold of 0.8 yields a substantial improvement with 95.6% of R epochs correctly identified, compared to 85.3% without a threshold. At this threshold, 323 participants retain at least 5 min of high-confidence REM sleep, while 13 do not.

The usefulness of confidence estimates was assessed by applying confidence thresholds to exclude epochs with uncertain sleep staging from further analysis. The left panel of Fig. 7 shows the percentage of participants in the combined PACE and CBC datasets who retain at least 10, 30, or 50 predicted REM epochs during a single overnight polysomnogram as the confidence threshold increases. As the confidence threshold increases, recall decreases and precision increases, while a larger proportion of subjects are unable to meet the recommendation of at least 5 min of REM sleep for subsequent RSWA evaluation^17^. With a threshold of 0.8, precision and recall were 0.96 and 0.89, respectively. Raising the threshold to 0.9 yielded precision and recall of 0.98 and 0.86. Thresholds above 0.9 further improve precision with a marked decline in recall. Corresponding REM sleep confusion matrices are presented in Fig. 7 (right panel). Similar associations between REM sleep confidence, recall and precision were observed across all clinical subgroups (Fig. S8).

Confidence-based thresholding produced different effects in the pretrained and generalized models (Fig. S9). In addition to higher baseline REM sleep precision, the generalized model showed numerically larger gains in precision after thresholding than the pretrained model, while preserving sufficient REM sleep in more subjects. In the PACE + CBC dataset, applying a threshold of 0.8 increased REM sleep precision by 10.3 percentage points using the generalized model, compared with 9.4 percentage points with the pretrained model, despite having a higher precision before thresholding (85.3% vs. 82.2%) and retaining ≥5 min of REM sleep in more subjects (323 vs. 302). Similar patterns were observed in the DCSM dataset.

## Discussion

Applying the *Pretrained Model* directly to datasets comprising patients with PD and iRBD substantially reduced performance (*κ* = 0.66) compared with multicenter datasets of non-neurodegenerative participants (*κ* = 0.82). Similarly, another study found substantially reduced accuracy with the Pretrained Model in early PD^32^. Fine-tuning the Pretrained Model on neurodegenerative datasets produced the *Generalized Model* with markedly improved agreement (*κ* = 0.74, *p* < 0.001). Total sleep time, sleep efficiency, and sleep onset latency were not significantly different when comparing the generalized model with human staging, but the proportion of N1 sleep and wakefulness after sleep onset–both of which are notoriously difficult to stage–was slightly higher. Although fine-tuning did not eliminate differences in sleep architecture metrics, these appeared small and generally diminished with fine-tuning.

A previous study of interrater agreement in PD reported *κ* = 0.62, with N1 showing the lowest agreement. Higher age and OSAS were identified as predictors of reduced agreement^25^. Similarly, the Generalized Model showed its weakest performance in N1 and a significant negative association between *κ* and both age and AHI (*p* < 0.001). These parallels likely reflect intrinsic challenges and variability in human scoring, which limit the model’s ability to learn generalizable rules, particularly for N1, and impose a ceiling on achievable performance. Model performance clearly exceeding human interrater agreement would raise concerns about overfitting.

U-Sleep has been shown to robustly reproduce AASM scoring in non-neurodegenerative populations^30^. However, performance may be reduced in new patient populations, such as those with neurodegenerative diseases, in which AASM rules may be applied differently. Possibly, the model may have difficulties with rules based on the absence of specific features–for example, scoring REM in epochs preceding REM (rule I3) or continuing N2 scoring without definitive N2 phenomena (K-complex or spindle, rule G4). This challenge may be especially pronounced in patients with neurodegenerative disease, in which rapid eye movements may be absent, and N2 phenomena are sparse. On average, the Generalized Model began scoring REM sleep and N2 ≈0.5 epochs earlier and continued slightly longer than human raters, resulting in increased durations of 1.18 and 0.84 epochs for REM sleep and N2, respectively. Several factors may explain this deviation. First, the model is less responsive to sleep stage transitions (Table 1, Fig. S3), generally producing longer stage durations before switching, possibly due to lower sensitivity to EEG changes associated with arousals or to inconsistent manual staging of short transitions, which makes these difficult to predict. Second, the model assigns sleep stages at sample-rate resolution (128 Hz), independent of the human epoch division, which is applied post hoc. Rules that rely on epoch boundaries–for example, scoring N2 in the next epoch when a definite N2 phenomenon occurs in the last half of the current epoch–are therefore difficult for the model to reproduce, potentially biasing N2 onset earlier. Notably, the model also appears to adopt ‘backward scoring’ of REM sleep even when REM sleep features are sparse. Overall, these deviations in N2 and REM scoring appear minor, as both stages maintain very high accuracy.

Site-Specific Models yielded only marginal improvements, significant only in the CBC dataset. In the clinical hold-out dataset from DCSM, post hoc finetuning also failed to improve overall *κ* or stage-wise F1 compared to the Generalized Model. Together, these findings suggest that the Generalized Model, jointly fine-tuned on PACE and CBC, is close to the performance ceiling for U-Sleep in these cohorts. Notably, the performance either exceeds or resembles previously reported human interrater κ in PD^25,26^. A Generalized Model also offers practical advantages. Combining datasets from multiple sites and raters reduces the risk of overfitting to specific raters or local data patterns, thereby markedly improving accuracy when applied to new sleep clinics. It removes the need for fine-tuning and revalidation at each new site, and extensive fine-tuning on specific neurodegenerative data might otherwise compromise generalizability to controls or other sleep disorders–an effect not observed for the current model.

Clinically, a model must be robust both in the presence and absence of the target disease and its comorbidities. The Generalized Model demonstrated such robustness across iRBD and early-to-moderate PD, with no significant differences in per-night *κ* value between clinical subgroups and with no clear association between *κ* value and disease duration in our early-to-moderate PD patients. In the hold-out clinical dataset from DCSM, staging performance increased markedly with the Generalized Model, but a Site-Specific model showed only marginal, non-significant improvements. Notably, REM staging accuracy across all datasets did not differ between clinical subgroups. This is encouraging if the model is to be applied in automated RBD diagnostics. In contrast, N1 and N3 accuracy were significantly reduced in early-to-moderate PD compared to controls, particularly in PD^+RBD^. This subgroup exhibits more widespread cortical pathology, which may lead to secondary EEG alterations, thereby complicating human and automated staging of N1 and N^33^.

In PACE and CBC, control participants were recruited for iRBD screening and therefore exhibited risk factors, such as self-reported dream enactment or nocturnal movements. This control group reflects the population encountered in clinical settings and ensures comparable age and sex distributions across groups. The higher AHI in controls likely reflects obstructive sleep apnea (OSA)- related movements that mimic RBD behavior, leading to often undiagnosed OSA patients participating in RBD screening. Such participants, particularly when undiagnosed, may lead to false positives in machine-learning-based prospective screening studies, especially if they are not included in the training datasets. Although a healthier control group might have produced higher staging agreement, the present controls provide a more realistic representation of non-neurodegenerative patients encountered in clinical screening, including subjects with undiagnosed OSA. In the future, a generalized model could be extended by incorporating datasets from other neurodegenerative diseases, such as mild cognitive impairment, DLB, and Alzheimer’s disease, to further enhance applicability across the clinical spectrum.

To explore patterns underlying the lowest staging performance, all recordings with *κ* < 0.6 were selected for blinded re-assessment, along with 10 randomly chosen recordings with *κ* > 0.6. In the high-agreement group, rater 1 (R1) and the second, blinded rater (R2) showed substantial agreement across all recordings, consistent with previously reported interrater values^25^. As expected, most disagreement occurred in N1 and N3. N1 typically shows the lowest interrater agreement^19^, and the discrepancies in N3 may reflect EEG abnormalities during NREM sleep in PD^23^. A similar pattern emerged when comparing the second, blinded rater (R2) with the Generalized Model (G), suggesting that when the model agrees well with one rater, comparable agreement can be expected when introducing a new rater. Conversely, for low-agreement recordings (*κ* < 0.6), the interrater agreement between R1 and R2 was *κ* = 0.49, slightly lower than the agreement of each rater with the model. This indicates that such recordings were difficult to stage for both human raters and that, in these ambiguous cases, the model may represent a balanced compromise between differing interpretations of the AASM rules. This interpretation is supported by a strong linear relationship between *κ*(R1 vs G) and *κ*(R1 vs R2) (Fig. 4), implying shared sources of disagreement between model and raters. Interestingly, the model tended to align more closely with R1 in high-agreement cases and more with R2 in challenging cases. This pattern likely reflects *regression toward the mean*: a recording with an unusually low κ when assessed by the first rater tends to regress toward the mean (a less extreme value) when reevaluated by the second rater. Consequently, recordings with unusually low model agreement tend to show slightly higher agreement with a new rater, and vice versa. Additionally, unlike human raters, the model is deterministic, providing a stable reference that may be advantageous in clinical workflows involving multiple scorers.

In an extensive interrater study comparing two European centers and the Stanford-STAGES algorithm, κ between human raters was 0.66 in sleep-clinic patients, decreasing slightly to 0.61 when comparing human scoring to the algorithm, with the lowest agreement in N1 and N3^34^. As our datasets mainly consist of elderly patients with neurodegenerative diseases and were selected based on κ, direct comparison is not possible. Nevertheless, our findings demonstrate that although the model was fine-tuned using data partly scored by R1, it does not appear overfitted, as agreement with the unseen rater (R2) remained similarly high.

Common challenges in human sleep staging such as advanced age, OSA, and neurodegeneration are well recognized, whereas other deviations, like poor signal quality, are rarely documented. All of these represent potential pitfalls that sleep staging models must handle. Among these challenges, the Generalized Model showed similar vulnerabilities with lower accuracy associated with higher age and AHI. However, using AHI to predict automated accuracy is impractical, as AHI is derived from human scoring. Although age correlated with κ, this effect diminished after accounting for U-Sleep confidence, suggesting that EEG changes related to age are partly reflected in the model’s confidence estimates. In fact, when limiting predictors to those available without manual input, the U-Sleep confidence emerged as the only significant predictor of *κ* (*p* < 0.001).

U-Sleep has previously been applied in patients with chronic insomnia and pediatric populations^35,36^. Beyond standard sleep staging, U-Sleep can be adapted to specific purposes. A recent paper used U-Sleep confidence to derive measures of global and stage-wise uncertainty and sleep-stage mixing. These novel measures were associated with poorer motor and cognitive scores and faster motor decline, suggesting that relevant biomarkers may be derived from U-sleep models. In sleep staging, we demonstrate a strong linear relationship between confidence and staging accuracy, particularly during REM sleep, indicating that U-Sleep confidence provides a reliable measure of model trustworthiness. Clinically, confidence values could help identify recordings or segments warranting manual review, or flag data of questionable quality in retrospective datasets. Notably, two recordings showed low to moderate confidence (0.70 and 0.76) and low agreement with R1 (*κ* = 0.26 and *κ* = 0.32). At reassessment by R2, *κ* increased by 0.24 and 0.23, respectively, corresponding more closely with the model’s confidence and illustrating how confidence can predict interrater variability. Furthermore, stage-wise confidence can be extracted at any time scale, e.g., from 30-s epochs for sleep staging to 3-s mini-epochs for RSWA quantification, or even at finer resolutions for microsleep analysis.

We tailored the model for optimized REM sleep staging using confidence-based thresholding. For instance, applying an arbitrary cut-off of 0.8 increases REM sleep precision from 85 to 95.6%. Compared with the pretrained model, the generalized model achieved larger precision gains after thresholding while preserving sufficient REM sleep duration in more subjects, with 96% of subjects still having sufficient REM sleep duration to enable quantification of RSWA^17^. Accurate identification of REM sleep without unnecessary exclusion of subjects is important, as automated RSWA detection algorithms, such as RBDtector, have shown promising performance but rely heavily on precise REM sleep staging^37,38^. Unlike human raters, U-Sleep provides confidence estimates, enabling automated and unbiased selection of REM epochs. By default, all manually scored REM epochs are assumed to be equally reliable. To address this, RSWA quantification guidelines recommend re-staging REM sleep, focusing on archetypal REM sleep without arousals, N2 phenomena, and prolonged absence of REMs. This process is time-consuming and requires expertise^17^, and a direct comparison will be needed to examine the extent to which automatically selected REM sleep, based on different thresholding approaches, overlaps with manual re-staging and affects RSWA quantification. Similar confidence-based adaptations could be implemented for other stages or temporal resolutions with little additional effort. Another application of confidence estimates is identifying uncertain epochs throughout the night, such as those affected by arousals. Comparing mean confidence values between epochs with and without manually annotated arousals revealed significantly lower confidence in arousal-affected epochs across all sleep stages (*p* < 0.001). This finding suggests that applying a confidence threshold could help automatically exclude epochs containing arousals–or, conversely, target them for review in disorders characterized by frequent arousals, such as OSAS and insomnia. Furthermore, U-Sleep confidence could serve as an informative input feature for other machine learning models.

In diagnosing RBD, non-REM sleep stages are of limited importance, whereas distinguishing W and R is critical. In patients with dream enactment, human raters often use video to aid in the differentiation of stage R and W, and even with all available tools some R epochs remain difficult to stage due to debatable arousals, movements or artefacts. Algorithms may be vulnerable to misclassifying such pathological R epochs as W due to the presence of movements and artefacts that in healthy controls are almost exclusively seen during W. This tendency may explain why a previous random-forest classifier for automated sleep staging in iRBD demonstrated a high *κ*-value (0.73) and R-agreement (84%) in elderly HC, whereas performance deteriorated in RBD patients (*κ* = 0.54, R-agreement = 42%)^31^. Similarly, another automated approach achieved a sensitivity of 0.91 for staging R in HC, but only 0.56 in iRBD patients^39^. After fine-tuning, we reach *κ* = 0.74 and stage R F1-scores of 0.86 in iRBD patients, and we detect no differences in stage R F1-scores between controls and patients with RBD, suggesting that the fine-tuned model is relatively unaffected by the movements, artefacts or other challenges to automated sleep staging observed during R in patients with RBD, although some misclassification of R due to dream enactment cannot be excluded since video-evaluations were not available for this study.

As current diagnostic guidelines for RBD require vPSG documentation of dream-enactment behaviors, automated sleep staging would still need to be combined with review of synchronized video recordings, potentially supported by automated methods for detecting dream-enactment behaviors, such as video-based motion analysis or wrist actigraphy. Automated computer vision for analysis of vPSG video recordings can detect movements during manually annotated REM sleep in iRBD patients under standardized conditions, as shown in a large dataset of iRBD patients and healthy controls^40^. Similarly, wrist-worn actigraphy has shown promising results in detecting iRBD using multiple nights of recordings, and as current actigraphy analyses are generally agnostic to sleep stages, their performance may further improve by combining them with automated sleep staging. Such novel methods are promising ways of detecting dream enactment, but they are not currently recognized as substitutes for manual inspection of video recordings.

This study has several limitations. We focused on iRBD and early-to-moderate PD, as these groups are clinically relevant for early RBD detection. Model accuracy may be lower in more advanced PD, where EEG abnormalities are more pronounced, and staging becomes increasingly complex^27^. Patients with moderate-to-late PD were included in the DCSM dataset, although clinical data were limited. The datasets almost exclusively comprise Northern European, Caucasian participants, which may restrict generalizability to more diverse populations. Additionally, the generalizability of the model may be dependent on the type and manufacturer of the PSG equipment used for recording and affected by known or unknown variances in how different healthcare systems collect, analyze and interpret their polysomnographic data. The interrater experiment used a relatively small sample of recordings, selected based on *κ*, and therefore cannot be used to infer interrater agreement across the datasets. We did not perform formal assessments of inter-site or inter-rater scoring agreement in addition to the interrater experiment. To confirm the robustness of the models, broader cohorts encompassing later stages of PD and other neurodegenerative disorders, as well as more diverse populations across disease severity and demographic variation would be needed. To reach a diagnosis of RBD aided by automated sleep staging, future studies would need to investigate how to incorporate RSWA quantification and reliable detection of dream enactment using video, microphone or other methods.

We demonstrate that U-Sleep achieves strong generalizability across diverse datasets, including patients with neurodegenerative disorders and elderly controls with co-morbid sleep disturbances, as well as an independent clinical dataset from an unseen site. The model can be flexibly adapted for specific purposes such as REM detection, RSWA quantification, or other stage-specific analyses. For sleep staging algorithms to become clinically relevant, cross-site and cross-population robustness is essential to avoid the need for finetuning of site-, disease-, or purpose-specific models. Generalizable models, by contrast, enable consistent evaluation of large, multi-site datasets previously scored by different raters. Ultimately, automated and standardized sleep staging represents a crucial step toward scalable, time-efficient sleep diagnostics in an era of growing clinical demand and expanding screening initiatives.

## Methods

### Datasets

A publicly available, multisite dataset (PUB) comprising nearly 15,000 subjects and more than 19,000 polysomnograms from 12 sites with a diverse range of individuals, including adults and children, various sleep disorders, and healthy individuals. An overview of the datasets is provided in Table S1. All recordings include manually annotated sleep stages adhering to either the R&K or the AASM manual^41,42^. This dataset closely resembles the one used to train the U-Sleep model in Perslev et al.^28^, except that all polysomnograms from DCSM were excluded to prevent the same individual from appearing in both the pretraining and hold-out testing sets. The datasets in PUB were acquired from third-party databases and handled according to their data use policies.

Two research datasets with PSGs, collected at the Lundbeck Foundation Parkinson’s Disease Research Center at Aarhus University Hospital (PACE) and the Cologne-Bonn Cohort at the University of Cologne and University of Bonn (CBC), were included from ongoing studies. Both datasets include patients with iRBD, PD with RBD (PD^+RBD^), or PD without RBD (PD^-RBD^). Control subjects in both datasets were identified as research participants without neurodegenerative disease undergoing PSG without signs of RBD. Most controls were included in previously published studies of community-based screening for iRBD and were not specifically matched to the clinical subgroups^8,9^. All video-polysomnographies (vPSG) were performed and manually annotated in accordance with the AASM manual. RBD was diagnosed based on the ICSD-3 diagnostic criteria. All participants with available PSG were included in training, but participants were excluded from testing and downstream analysis in cases with inconclusive diagnosis, as these could not be assigned to a clinical subgroup. These cases were PD patients with a normal dopamine scan and non-PD participants with symptoms suggestive of dream enactment, but with an inconclusive vPSG.

Subject demographics and subgroup composition are shown in Table 4. All subjects provided informed written consent according to the Declaration of Helsinki. The studies were approved by the Central Denmark Region Committees on Health Research Ethics (1-10-72-160-16) and by the ethics committee of Cologne University Hospital (19-1408).

**Table 4:** Overview of datasets, their demographic data and sleep metrics for the human sleep scoring

	Training						Hold-out		
	PACE			CBC			DCSM		
	Control	iRBD	PD	Control	iRBD	PD	Control	iRBD	PD
**Demographics**									
N	15	36	83	74	102	29	87	36	81
Age (years)	70.0 [65-73]	66.5 [61-70]	65.0 [60-70]	62.0 [55-70]	67.0 [61-72]	69.0 [56-72]	52 [36-64]	65 [49-69]	67 [62-71]
Sex (male/female)	12/3	27/9	54/26	55/19	87/15	21/8	47/40	26/10	54/27
Body mass index (BMI, kg/m^2^)	26.2 ± 4.3	25.5 ± 4.1	25.9 ± 4.3	25.7 ± 3.0	25.7 ± 3.1	25.7 ± 3.7	25.8 ± 5.0	26.3 ± 5.2	25.2 ± 3.4
Apnea-hypopnea index (AHI, events/h)	20.8 ± 17.7	10.4 ± 11.3	14.7 ± 14.9	13.5 ± 13.8	7.9 ± 9.7	7.5 ± 6.6	6.9 ± 7.9	13.4 ± 16.2	10.9 ± 16.2
REM sleep behavior disorder (yes/no/NA)	0/15/0	36/0/0	36/44/3	0/74/0	102/0/0	16/13/0	87/0	0/36	51/30
Duration of RBD symptoms at PSG (years)		5.2 ± 4.3	5.3 ± 5.2		12.2 ± 6.6				
Time since PD diagnosis PSG (years)			1.5 ± 1.6			6.6 ± 4.4			
Hoehn and Yahr stage (1/2/3)			24/48/5						
MDS UPDRS-III motor score (ON medication)			19.3 ± 6.9						
MDS UPDRS-III motor score (OFF medication)		7.4 ± 7.2	17.9 ± 8.0		5.3 ± 3.4	35.4 ± 11.1			
Levodopa equivalent daily dose (mg/day)			226.9 ± 288.9			640.9 ± 431.9			
Epworth Sleepiness Scale score	5.0 ± 2.5	5.4 ± 3.0	6.5 ± 4.1	7.8 ± 3.5	7.2 ± 3.7	10.0 ± 4.3			
RBD Screening Questionnaire (RBDSQ) score	2.0 ± 1.4	10.1 ± 1.8	4.9 ± 3.1	7.8 ± 3.0	9.5 ± 1.8	5.4 ± 3.5			
Sniffin’ Sticks 12-item identification score					6.5 ± 2.6				
Sniffin’ Sticks 16-item identification score	7.7 ± 3.8	8.5 ± 2.8	7.5 ± 3.1	11.1 ± 2.6		7.3 ± 2.8			
Montreal Cognitive Assessment (MoCA), score	27.5 ± 2.1	27.1 ± 2.6	27.6 ± 1.8	28.2 ± 0.8	26.9 ± 2.2	26.5 ± 2.4			
**Sleep features**									
Total Sleep Time (min.)	370.8 ± 77.2	424.4 ± 74.3	394.1 ± 64.5	394.4 ± 70.1	395.4 ± 64.2	394.4 ± 58.3	411.3 ± 58.1	360.9 ± 76.0	336.6 ± 73.5
Sleep Efficiency (%)	72.9 ± 12.7	83.8 ± 8.4	76.9 ± 11.2	82.6 ± 10.3	81.9 ± 10.2	82.9 ± 10.0	87.9 ± 9.8	80.8 ± 10.4	76.6 ± 12.7
Sleep Onset Latency (min.)	9.6 ± 7.3	17.0 ± 15.1	11.1 ± 15.3	9.8 ± 10.9	11.7 ± 13.2	6.9 ± 6.0	8.0 ± 10.1	6.7 ± 8.2	5.2 ± 6.2
REM Latency (min.)	127.2 ± 69.0	136.7 ± 102.7	152.1 ± 83.7	104.7 ± 68.2	113.5 ± 77.4	115.6 ± 69.3	89.5 ± 53.4	137.2 ± 83.3	147.4 ± 105.8
N1 sleep fraction (% of total sleep time)	25.6 ± 16.6	16.4 ± 7.0	10.8 ± 7.4	22.0 ± 14.0	20.2 ± 9.9	15.5 ± 7.7	10.3 ± 8.6	15.3 ± 12.7	15.5 ± 12.4
N2 sleep fraction (% of total sleep time)	47.5 ± 12.7	46.8 ± 7.9	56.9 ± 8.1	48.8 ± 11.1	47.8 ± 9.0	48.1 ± 9.3	51.7 ± 9.7	50.8 ± 13.5	53.1 ± 17.5
N3 sleep fraction (% of total sleep time)	11.1 ± 5.8	17.9 ± 7.0	16.3 ± 8.7	15.2 ± 7.9	16.2 ± 6.6	15.1 ± 7.2	17.1 ± 10.6	16.2 ± 14.3	18.2 ± 15.7
R sleep fraction (% of total sleep time)	15.8 ± 6.6	18.9 ± 7.4	16.0 ± 5.7	14.1 ± 5.7	15.9 ± 5.5	21.4 ± 9.8	20.9 ± 6.6	17.6 ± 8.4	13.2 ± 10.0
Wake After Sleep Onset (min.)	129.3 ± 73.0	64.9 ± 39.1	105.3 ± 60.6	68.9 ± 46.0	68.8 ± 48.6	76.3 ± 55.1	51.0 ± 52.6	74.6 ± 44.0	97.8 ± 59.5
Stage changes (n)	171.7 ± 90.7	172.9 ± 63.7	144.4 ± 52.5	148.7 ± 48.2	136.7 ± 45.3	126.9 ± 48.4	119.7 ± 55.1	115.7 ± 49.6	97.2 ± 42.4

Controls from the DCSM dataset are sleep-clinic patients without a clinical diagnosis of PD and no evidence of RBD on vPSG. Controls from CBC and PACE are research participants, mostly participants undergoing screening for iRBD, who did not have a diagnosis of a neurodegenerative disorder and no evidence of RBD on vPSG. Data are presented as mean (SD), median [IQR], or n, as appropriate.

A clinical hold-out dataset was included from the Danish Centre for Sleep Medicine (DCSM) comprising patients undergoing diagnostic vPSG in the sleep clinic. This dataset is enriched with patients with iRBD and PD, some of whom have PD at an advanced stage. Controls are sleep clinic patients without evidence of RBD and without a clinical diagnosis of PD. The sleep clinic controls were not matched to the patients. Subject demographics and subgroup composition are shown in Table 4. The DCSM dataset had been anonymised prior to the present study under a general approval granted by the Danish Data Protection Agency^28^.

### Data preprocessing and Record rejection

All preprocessing was performed with our existing U-Sleep pipeline^43^. In brief, all PSG recordings were resampled to 128 Hz, scaled and outlier-clipped, and then high-pass filtered at 0.1 Hz. R&K sleep-stage annotations were converted to AASM by collapsing N3 and N4. All non-scored epochs were annotated as a separate “unknown” class, and these were not included in cost calculation or model evaluation.

For the finetuning and “hold-out” datasets, all nights were limited to their lights-off/lights-on periods during training, validation, and testing. Recordings with less than three hours of sleep (12 recordings) or with more than 20% unscored epochs (11 recordings) were rejected. All sleep epochs containing more than 50% flatline were also labeled as “unknown”. Additionally, recordings with all EEG and EOG channels flatlining simultaneously for more than 10% of the recording were rejected (8 recordings). A segment of the recording was classified as a flatline if the signal had a peak-to-peak amplitude of less than 10^−8^ volts, evaluated over 30-second windows. After rejections, the datasets used for training the sleep staging models consisted of 215 eligible recordings in CBC and 134 in PACE, while 204 recordings from DCSM remained for hold-out testing. The final test results presented excluded an additional 10 recordings from CBC due to the lack of a conclusive PD and RBD diagnosis. This is summarized in Table S4.

### Sleep staging

**Fig. 8:**
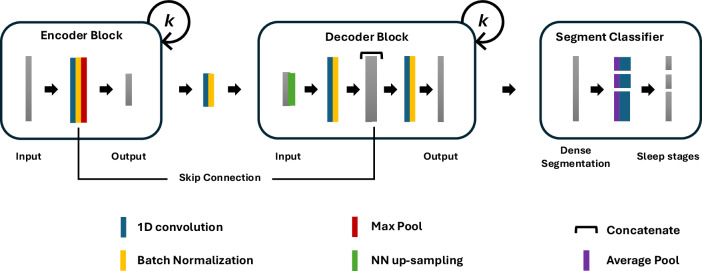
U-Sleep model architecture. Adapted from Perslev et. al 2021 (Open Access under Creative Commons License 4.0 http://creativecommons.org/licenses/by/4.0/).

**Fig. 9:**
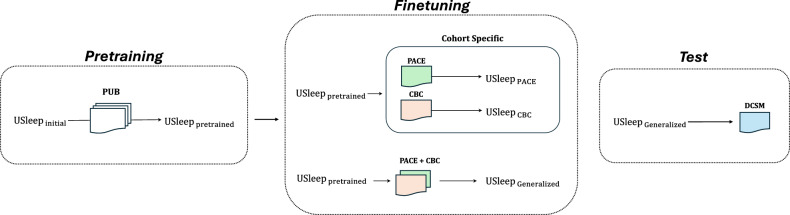
Overview of the model training setups. The Pretrained Model was fine-tuned in three ways: two specialized for specific cohorts (PACE and CBC), and the third trained on both simultaneously to obtain the Generalized Model. Finally, the Generalized Model was tested on the clinical hold-out dataset DCSM.

U-Sleep is a fully convolutional neural network used for automatic sleep staging. It processes temporal data directly and was originally implemented as a 2-channel model with one EEG and one EOG channel, as described by Perslev et al.^28^. U-Sleep consists of three parts: an encoder, a decoder, and a segment-classifier, as shown in Fig. 8. The encoder consists of *k* encoder layers, each comprising a 1D convolution, Batch Normalization, and a max pooling layer. The output of the encoder is a feature representation of the original signal with lower temporal resolution. This is given as input to the decoder, which consists of *k* decoder layers. Each layer consists of an up-sampling step and two layers of 1D convolution and batch normalization. Between these two layers, a skip connection is also given, originating from its corresponding encoder block. The decoder’s output is a sleep-stage segmentation with the same temporal resolution as the original signal. This is provided as input to the segment classifier, which averages across 30-second sleep epochs to produce the final sleep stage classification. The high-level overview of the training setup is shown in Fig. 9. A Pretrained Model was obtained by training on large multisite datasets. The Pretrained Model was then fine-tuned on PACE and CBC, separately and combined, resulting in Site-Specific Models and a Generalized Model. The Generalized Model was tested on the clinical hold-out DCSM dataset.

A two-channel U-Sleep model with one random EEG and one random EOG channel from the available PSG was used for all training setups. We used an encoder/decoder depth of 12. U-Sleep has two other hyperparameters: progression factor and complexity factor, which were set to 2 and 1.67, respectively, as in Perslev et al.^28^. This setup resulted in ~3.1 million trainable parameters. During training, U-Sleep was shown minibatches of 35 sleep epochs, each consisting of a single EEG and EOG channel combination. These combinations were sampled from the data using a semi-random sampling technique similar to that in Perslev et al. We used a cross-entropy loss function, the Adam Optimizer and a batch size of 64. During testing, the model was shown all possible combinations of a single EEG and EOG channel from the given PSG recording. The results from each forward pass were summed, and the majority class was determined for each sleep epoch.

For pretraining the PUB dataset was split into three subsets on a per-site-per-subject basis: training, validation, and test, with subject-based splits of 0.94, 0.03, and 0.03, respectively. Due to the random sampling technique, each epoch consisted of 886 minibatches. The initial learning rate was 10^−4,^ with a decaying scheduler, patience of 40 epochs, and a down-scaling factor of 0.5. Early stopping was applied with a patience of 80 epochs, without any validation improvement.

All fine-tuned models were evaluated using 10-fold cross-validation at the per-dataset, per-subject level. Ten subjects were set aside as validation subjects for each split. The learning rate was set at a fixed value of 10^−5^. Each cross-validation split ran for up to 50 epochs, with early stopping at 10 epochs. Each epoch consisted of 50 mini-batches. For testing the Generalized Model on DCSM, we combined all models from the finetuning process into an ensemble, resulting in a per-model majority-vote sleep-stage prediction. DCSM served as a “hold-out” dataset, and no aspects of model architecture, hyperparameter-tuning or model selection were influenced by DCSM data inspection or sleep-staging performance.

### Sleep staging analysis

When possible, Cohen’s κ was preferred to evaluate sleep staging performance, and F1-scores were used for comparability with previous publications and to evaluate stage-wise performance. Sleep metrics were calculated for both human and automated sleep scorings using commonly used definitions (Table S8). The metrics for each model type were calculated and compared with the human labels. Independent or pairwise permutation tests were used to determine significance, depending on the nature of the data. When evaluating Cohen’s *κ* or F1 scores, it was always done on a per-night basis unless otherwise specified.

To investigate U-Sleep’s ability to correctly identify length and latency of REM sleep and N2 sleep periods, an analysis was done on start-offset and length-offset of such sleep periods. First, all REM cycles lasting for more than five epochs in both the human and automatic scoring were identified. All automatically and manually scored cycles with a single overlapping counterpart were then identified and interpreted as representing the same underlying REM sleep cycle. Finally, the starting point offset and length differences for these cycles were calculated. The Shapiro-Wilk test was used to assess normality.

### Interrater assessment

PSG recordings for the interrater assessment were chosen based on a lower-bound *κ* value of 0.6. An additional ten records with an initial *κ* value above 0.6 were selected (five from PACE, five from CBC). These were randomly sampled from five equal-sized intervals between 0.6 and 0.9 to represent the full range of performance adequately. The threshold of 0.6 was chosen such that the number of recordings included did not exceed the human resources available for rescoring.

### Confidence estimates

Hypnodensities and model confidences were derived from intermediate model outputs. The model sleep staging output can be defined as a vector ${P}_{{sleep}}={{\mathbb{R}}}^{E}$, where *E* is the number of sleep epochs in the PSG recording. Meanwhile, model confidence can be expressed as ${P}_{{conf}}={{\mathbb{R}}}^{E\times S}$, where S is the number of stages, and ${{P}_{{conf}}}_{{ij}}$ is the model confidence of epoch *i* being the sleep stage *j*. Furthermore, the classification of an epoch *i* is given by ${P}_{{slee}{p}_{i}}={\rm{\arg }}\max ({p}_{{con}{f}_{i}})$. As such, ${P}_{{conf}}$ is a matrix that describes the model’s certainty and provides more insight into the decision boundaries.

### Linear Regression Model

Three types of linear mixed regression models were fitted with per-night Cohen’s *κ* score as the dependent variable. Cohen’s *κ* is bounded between −1 and 1. However, in our study, *κ* values were mainly in the range of 0.5-0.8 without approaching the boundaries, supporting the use of linear mixed effect models that assume a continuous, unbounded outcome. To account for the random variable of cohort, it was modeled as a random effect with a random intercept. All models (A, B and C) followed the same generic pattern, as shown in the equation below

$${\rm{Cohe}}{{\rm{n}}}^{{\prime}}{\rm{s}}\,\kappa \sim {fixed\;effects}+\left(1|{cohort}\right)$$

The fixed effects refer to additive terms defined by the variables listed in Table 3. A detailed definition of each variable is found in Table S10, while full model specifications are found in Table S11.

Model C specified in Table 3 was fitted and evaluated for all recordings in PACE and CBC using 10-fold cross-validation to determine performance and identify potential overfitting. The absolute residuals were averaged to find the Mean Absolute Error (MAE). Significant parameters were identified with two-tailed z-tests. A plot of the residuals is found in Fig. S6, showing an MAE of 0.052.

### Confidence and arousals

The confidence of an epoch *i* was determined as $\max \left({P}_{{con}{f}_{i}}\right)$. The overall mean confidence of a PSG recording was therefore determined as $\frac{\mathop{\sum }\limits_{i=0}^{E-1}\max \left({P}_{{con}{f}_{i}}\right)\,}{E}$, where E is the number of epochs in the PSG. Mean confidence for epochs affected by arousals and for epochs not affected by arousals was then calculated. An epoch was considered affected if it overlapped with the annotated arousal. Arousals were manually annotated during routine sleep staging in accordance with the AASM manual and automatically categorized into subtypes based on coinciding polysomnographic events. Arousals coinciding with respiratory events, including apnea, hypopnea, desaturation and snoring, were considered respiratory arousals.

### Confidence as a threshold for REM sleep classification

With no confidence threshold, an epoch is classified as R when ${\rm{\arg }}\max ({p}_{{con}{f}_{i}})=R$, with *R* being the index of the REM sleep stage. If we treat R classification as a two-class problem, we have a new matrix ${{P}_{{rem}}}_{i}\in {{\mathbb{R}}}^{S}$, which only contains two values–confidence of R and confidence of non-R. A confidence threshold is then introduced, such that REM sleep classification only occurs if ${P}_{{{rem}}_{i0}} < {P}_{{{rem}}_{i1}}$ and ${P}_{{{rem}}_{i1}} > T$, where *T* is a given threshold between 0 and 1.0. Epochs where ${P}_{{{rem}}_{i0}} < {P}_{{{rem}}_{i1}}$ and ${P}_{{{rem}}_{i1}} > T$ are treated as “unclassified” and do not appear in the confusion matrices.

## Supplementary information

Supplementary information

## Data Availability

The datasets used in the current study are not publicly available because they contain personal health data protected by local ethics and data-protection regulations. Access requires specific approvals, including data sharing agreements. Fully anonymized, processed data may be shared on reasonable requests, subject to necessary regulatory and contractual agreements.

## Code availability

The underlying code for the U-Sleep model is available at https://github.com/jesperstroem/U-Sleep-for-RBD-PD. The repository contains a detailed guide on how to set up and use the pipeline to sleep-stage various types of files containing PSG data using Python (PyTorch and PyTorch Lightning packages). The installation process is done in a few steps through a command line, and there are code examples available, such that only a few lines need to be adapted before use, meaning it requires only minimal Python experience to set up. Once set up, it is possible to use either the Pretrained Model or the Generalized model. Additionally, it is possible to customize which PSG channels are used for classification.
