# Supplementary material for: Fully-automated sleep staging for Parkinson’s disease and isolated REM sleep behavior disorder
Source: NPJ Digit Med. 2026 Aug 11;9:629. doi: 10.1038/s41746-026-02946-2 (PMC13476445; doi:10.1038/s41746-026-02946-2)
Supplement: Supplementary file 1 [file 41746_2026_2946_MOESM1_ESM.pdf]

Supplementary material

Dataset	Subjects	Records	Subject Composition
ABC	49	132	Adults with class II obesity and severe OSA
CCSHS	515	515	Children aged 8-11 years
CFS	730	730	2284 subjects across 361 families
CHAT	1 232	1 639	Children aged 5-10 years with OSA
HPAP	247	247	Adults with moderate to severe OSA
MESA	2 055	2 055	Multi-ethnic group aged 45-84 years
MROS	2 903	3 921	Men aged 65 or older
PHYS	919	919	Patients from MGH sleep center
SEDF-SC	76	145	Healthy adults aged 25-101
SEDF-ST	21	36	Adults under the influence of temazepam
SHHS	5 797	8 444	Adults aged 40 or older
SOF	453	453	Women aged 65-89 years
Total	14997	19236	

Table S1 *- Overview of the multi-site dataset (PUB) used to train the base U-Sleep model*

			**F1**					
**Cohort**	**Accuracy**	**Cohen's κ**	**Wake**	**N1**	**N2**	**N3**	**REM**	**Macro**
ABC	0.82 ± 0.02	0.76 ± 0.03	0.86 ± 0.02	0.60 ± 0.10	0.84 ± 0.02	0.75 ± 0.07	0.91 ± 0.05	0.79 ± 0.00
CHAT	0.89 ± 0.04	0.85 ± 0.05	0.95 ± 0.03	0.64 ± 0.09	0.87 ± 0.05	0.91 ± 0.05	0.90 ± 0.07	0.85 ± 0.04
CCSHS	0.90 ± 0.04	0.87 ± 0.06	0.97 ± 0.02	0.54 ± 0.19	0.89 ± 0.05	0.86 ± 0.08	0.91 ± 0.04	0.84 ± 0.06
CFS	0.87 ± 0.08	0.80 ± 0.11	0.94 ± 0.07	0.41 ± 0.17	0.85 ± 0.07	0.69 ± 0.24	0.83 ± 0.19	0.74 ± 0.10
HOMEPAP	0.82 ± 0.07	0.75 ± 0.09	0.88 ± 0.09	0.49 ± 0.17	0.83 ± 0.08	0.73 ± 0.24	0.89 ± 0.05	0.76 ± 0.09
MESA	0.88 ± 0.06	0.82 ± 0.09	0.95 ± 0.05	0.57 ± 0.13	0.87 ± 0.06	0.55 ± 0.31	0.89 ± 0.14	0.77 ± 0.09
MROS	0.89 ± 0.04	0.83 ± 0.07	0.95 ± 0.06	0.38 ± 0.16	0.87 ± 0.06	0.57 ± 0.27	0.86 ± 0.16	0.73 ± 0.09
SEDF-SC	0.88 ± 0.04	0.78 ± 0.07	0.98 ± 0.00	0.38 ± 0.12	0.69 ± 0.15	0.57 ± 0.22	0.80 ± 0.17	0.69 ± 0.06
SEDF-ST	0.86 ± 0.03	0.81 ± 0.04	0.86 ± 0.01	0.60 ± 0.18	0.87 ± 0.02	0.89 ± 0.01	0.89 ± 0.03	0.82 ± 0.05
SHHS	0.87 ± 0.07	0.81 ± 0.11	0.93 ± 0.08	0.47 ± 0.20	0.87 ± 0.08	0.71 ± 0.25	0.87 ± 0.19	0.77 ± 0.12
SOF	0.88 ± 0.05	0.82 ± 0.07	0.94 ± 0.04	0.38 ± 0.16	0.86 ± 0.05	0.80 ± 0.12	0.90 ± 0.06	0.78 ± 0.06
PHYS	0.83 ± 0.05	0.76 ± 0.07	0.79 ± 0.11	0.61 ± 0.12	0.86 ± 0.07	0.71 ± 0.26	0.86 ± 0.13	0.77 ± 0.08
**Mean**	**0.88 ± 0.06**	**0.82 ± 0.09**	**0.93 ± 0.08**	**0.48 ± 0.19**	**0.87 ± 0.07**	**0.68 ± 0.27**	**0.87 ± 0.16**	**0.77 ± 0.10**

Table S2 – *Mean and standard deviation of per-night Cohen's κ and per-night F1 scores when testing the Pretrained Model on the large multi-site dataset*

Cohort	Our model	Perslev et al.
ABC	0.81	0.77
CHAT	0.86	0.85
CCSHS	0.84	0.85
CFS	0.77	0.82
HOMEPAP	0.78	0.78
MESA	0.81	0.79
MROS	0.76	0.77
SEDF-SC	0.70	0.79
SEDF-ST	0.83	0.76
SHHS	0.80	0.80
SOF	0.79	0.78
PHYS	0.81	0.79
**Mean**	**0.80**	**0.80**

Table S3 – *Per-dataset Macro F1 scores for our Pretrained Model and the model reported by Perslev et al.*

		**Rejection reasons**					
**Site**	**Total Recordings**	**Less than 3 hours of sleep**	**More than 20% unscored epochs**	**More than 10% flatline in all channels simultaneously**	**Used for training**	**Inconclusive PD and RBD diagnosis**	**Used in testing, analysis and results**
PACE	136	1	1	0	134	0	134
CBC	231	3	5	8	215	10	205
DCSM	217	8	5	0	0	0	204
Total	584	12	11	8	553	10	543

Table S4 - *Overview of all recordings and the number of rejected recordings across datasets, as well as the reasons for rejection. Ten recordings from CBC were left out of the analysis and results due to an inconclusive PD and RBD diagnosis. DCSM was only used for testing and did not affect the training process in any way. PACE and CBC were used for both training and testing through cross-validation.*

**Cohort**	**Pretrained**	**Generalized**	**Site Specific**
PACE	0.65 ± 0.11<br>0.67 [0.59-0.72]	**0.72 ± 0.09***<br>**0.73 [0.67-0.79]***	0.72 ± 0.09<br>0.74 [0.67-0.79]
CBC	0.67 ± 0.09<br>0.69 [0.62-0.74]	**0.76 ± 0.08***<br>**0.76 [0.72-0.81]***	0.76 ± 0.08<br>**0.77 [0.73-0.81]***
DCSM	0.6 ± 0.17<br>0.64 [0.53-0.72]	**0.64 ± 0.18***<br>**0.69 [0.59-0.76]***	0.66 ± 0.18<br>0.7 [0.59-0.78]

Table S5 – *Mean ± SD and Median [IQR] of per-night Cohen's κ for each model type and each cohort. * indicates a significant improvement from the row and column directly to the left ($p < 0.05$).*

	Cohen's κ			F1 scores – Generalized					
Subgroup	Pretrained	Generalized	Site Specific	W	N1	N2	N3	REM	Macro
Control	0.67±0.09	0.75±0.09	0.75±0.09	0.82±0.11	0.59±0.12	0.84±0.09	0.81±0.15	0.87±0.12	0.78±0.07
iRBD	0.65±0.10	0.74±0.08	0.74±0.08	0.80±0.11	0.53±0.13	0.84±0.06	0.83±0.13	0.86±0.11	0.77±0.06
PD^{-RBD}	0.69±0.10	0.75±0.09	0.76±0.09	0.85±0.10	0.46±0.15	0.84±0.08	0.78±0.16	0.90±0.08	0.77±0.07
PD^{+RBD}	0.66±0.10	0.72±0.09	0.73±0.08	0.82±0.12	0.44±0.15	0.84±0.06	0.75±0.12	0.85±0.10	0.74±0.07

***Table S6 –** Mean and standard deviation of Cohen's κ values across models and clinical subgroups for the PACE and CBC cohorts. Additionally, the mean and standard deviation of F1 scores for the Generalized Model across clinical subgroups.*

Affected by arousal	Wake Confidence	N1 Confidence	N2 Confidence	N3 Confidence	REM Confidence	Global Confidence
**All arousals**						
No	0.83±0.07	0.58±0.05	0.82±0.05	0.82±0.05	0.84±0.08	0.77±0.12
Yes	0.75±0.08	0.57±0.05	0.73±0.06	0.69±0.08	0.75±0.09	0.70±0.10
**Respiratory arousals**						
No	0.83±0.07	0.58±0.05	0.81±0.05	0.83±0.05	0.84±0.07	0.77±0.12
Yes	0.76±0.08	0.56±0.05	0.73±0.07	0.69±0.08	0.75±0.09	0.70±0.10
**Non-respiratory arousals**						
No	0.83±0.06	0.58±0.04	0.81±0.05	0.81±0.06	0.84±0.06	0.76±0.12
Yes	0.73±0.09	0.58±0.05	0.74±0.08	0.69±0.08	0.76±0.10	0.70±0.11

***Table S7 –** Per-night mean confidence scores calculated for the epochs with and without arousals separately, for each arousal condition – all combined, respiratory arousals and non-respiratory arousals. For each condition, only PSG nights with at least 3 arousal-affected epochs were included, which explains the slight differences in non-affected values across conditions. For all conditions, there is a significant decline in confidence across all sleep stages when epochs are affected by an arousal ($p < 0.001$).*

Metric	Abbrev.	Description
Total Sleep Time	TST	The total amount of sleep in minutes.
Sleep Efficiency	SEFF	The fraction of sleep epochs compared to the total number of epochs.
Sleep Onset Latency	SOL	Minutes until the first sleep epoch.
REM Latency	REMLAT	Minutes until the first R epoch
N1 fraction	PN1	The fraction of time spent in N1 compared to the Total Sleep Time
N2 fraction	PN2	The fraction of time spent in N2 compared to the Total Sleep Time
N3 fraction	PN3	The fraction of time spent in N3 compared to the Total Sleep Time
R fraction	PREM	The fraction of time spent in R compared to the Total Sleep Time
Wake After Sleep Onset	WASO	Minutes spent in W after the first sleep epoch.
Stage changes	STAGEC	Total number of stage changes throughout the entire night.

***Table S8 -** Overview of sleep metrics calculated to review the performance of U-Sleep. All metrics are from the AASM manual except for Stage Changes.*

			F1				
**Model**	**Accuracy**	**Cohen's κ**	**Wake**	**Light Sleep**	**Deep Sleep**	**REM**	**Average**
Pretrained	0.83±0.06	0.72±0.10	0.74±0.13	0.86±0.05	0.73±0.20	0.79±0.19	0.78±0.09
Generalized	0.87±0.05	0.79±0.09	0.82±0.11	0.88±0.05	0.79±0.17	0.86±0.14	0.84±0.08
Site Specific	0.88±0.05	0.79±0.09	0.82±0.11	0.88±0.05	0.79±0.17	0.86±0.14	0.84±0.08

Table S9 - *Overall and stage-wise model performance of all three model types in a 4-stage classification setup. N1 and N2 have been merged to form the "Light Sleep" stage, and "Deep Sleep" represents N3.*

**Parameter**	**Type**	**Description**
Age	Pre PSG	Age of the subject in years.
Sex	Pre PSG	Sex of the subject (female/male).
BMI	Pre PSG	Body Mass Index of the subject.
PD	Pre PSG	Whether or not the subject is diagnosed with Parkinson's Disease.
AHI	Post PSG evaluation	Apnea-Hypopnea Index of the subject.
RBD	Post PSG evaluation	Whether or not the subject is diagnosed with REM Sleep Behavior Disorder.
Cohort	Post PSG evaluation	Which cohort the PSG recording belongs to (PACE or CBC)
Cohen's κ	Post U-Sleep staging	Agreement between the manual scorer and U-Sleep, expressed in Cohen's κ.
PN1 %	Post U-Sleep staging	Percentage of N1 sleep.
STAGEC #	Post U-Sleep staging	Absolute number of sleep stage changes throughout the night.
U-Sleep Confidence %	Post U-Sleep staging	Mean model confidence in the predicted class for each epoch.

Table S10 – *Detailed descriptions of each variable used to fit the linear mixed models. Both "Pre-PSG" and "Post PSG evaluation" refer to values at the time of the PSG recording. The "Post U-Sleep staging" variables all relate to the output of the Generalized Model.*

**Model**	**Dependent variable**	**Fixed effects**	**Random effects**	**Model specification**
A	Cohen's κ	Age, Sex, BMI, PD	Cohort	$Cohen's\ \kappa \sim Age + Sex + BMI + PD + (1\|cohort)$
B	Cohen's κ	Age, Sex, BMI, PD, AHI, RBD	Cohort	$Cohen's\ \kappa \sim Age + Sex + BMI + PD + AHI + RBD + (1\|cohort)$
C	Cohen's κ	Age, Sex, BMI, PD, PN1 %, STAGEC #, U-Sleep Confidence %	Cohort	$Cohen's\ \kappa \sim Age + Sex + BMI + PD + PN1\% + STAGEC\# + USleep\ Confidence\% + (1\|cohort)$

Table S11 – *Detailed model specifications for each fitted Linear Mixed Model (A,B and C). All models take the random effect of cohort into consideration by expressing it as a random intercept for each group.*

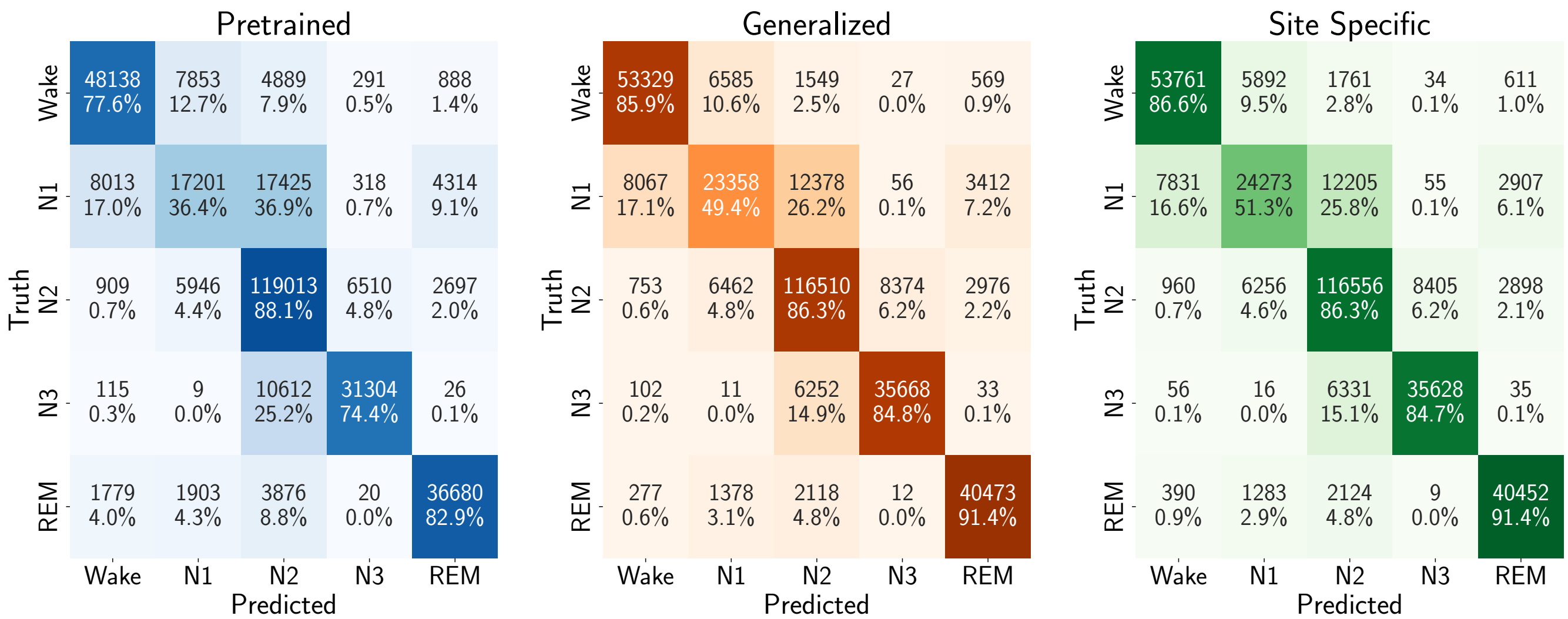

Figure S1 *- Confusion matrices for the Pretrained, Generalized and Site-Specific Models.*

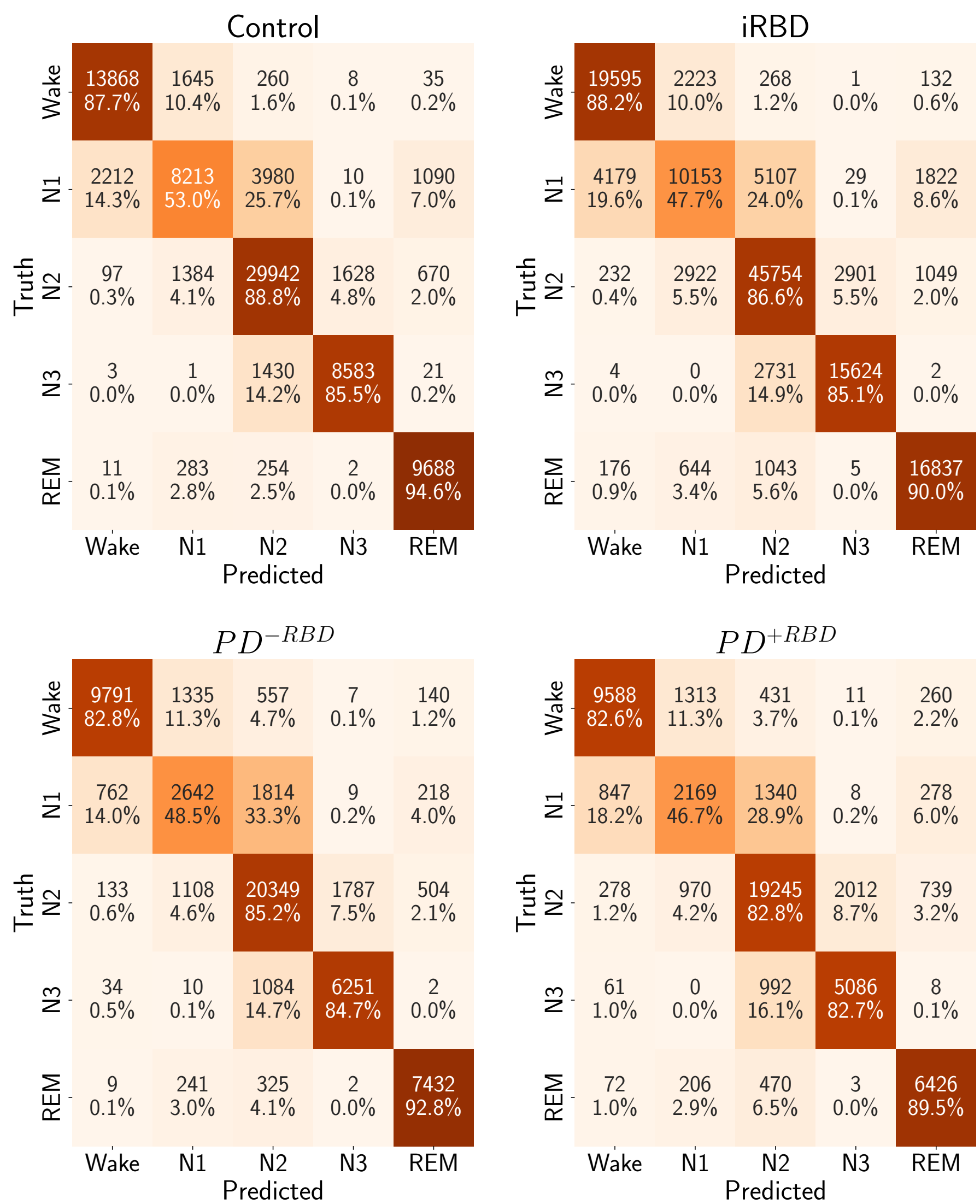

Figure S2 - *Confusion matrices for the Generalized Model across the four clinical subgroups.*

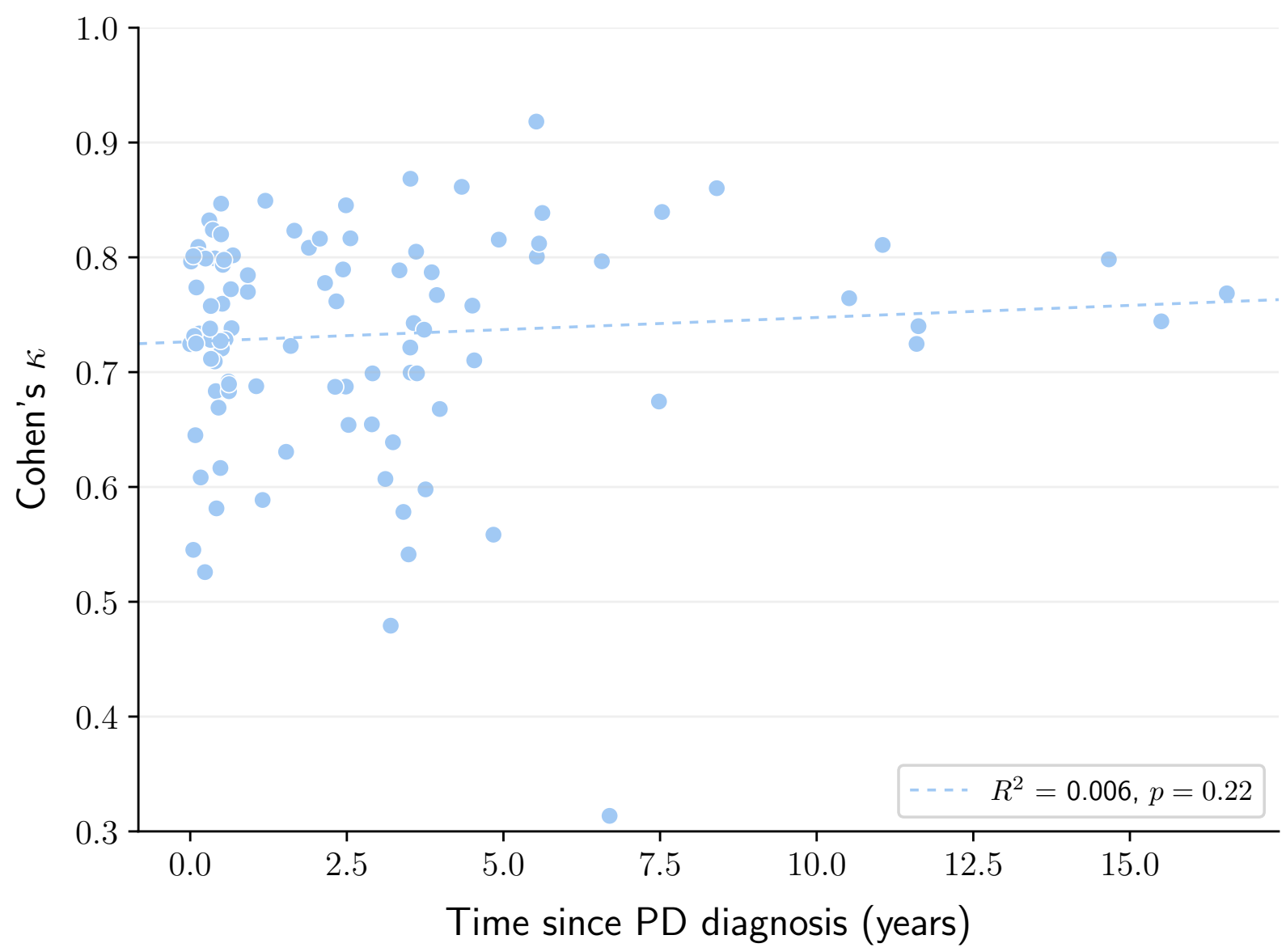

Figure S3 - *Cohen's κ as a function of disease duration (time since PD diagnosis) in the PD[-RBD] and PD[+RBD] subgroups using the Generalized model. A non-significant positive association (p = 0.22) is indicated by the dashed line.*

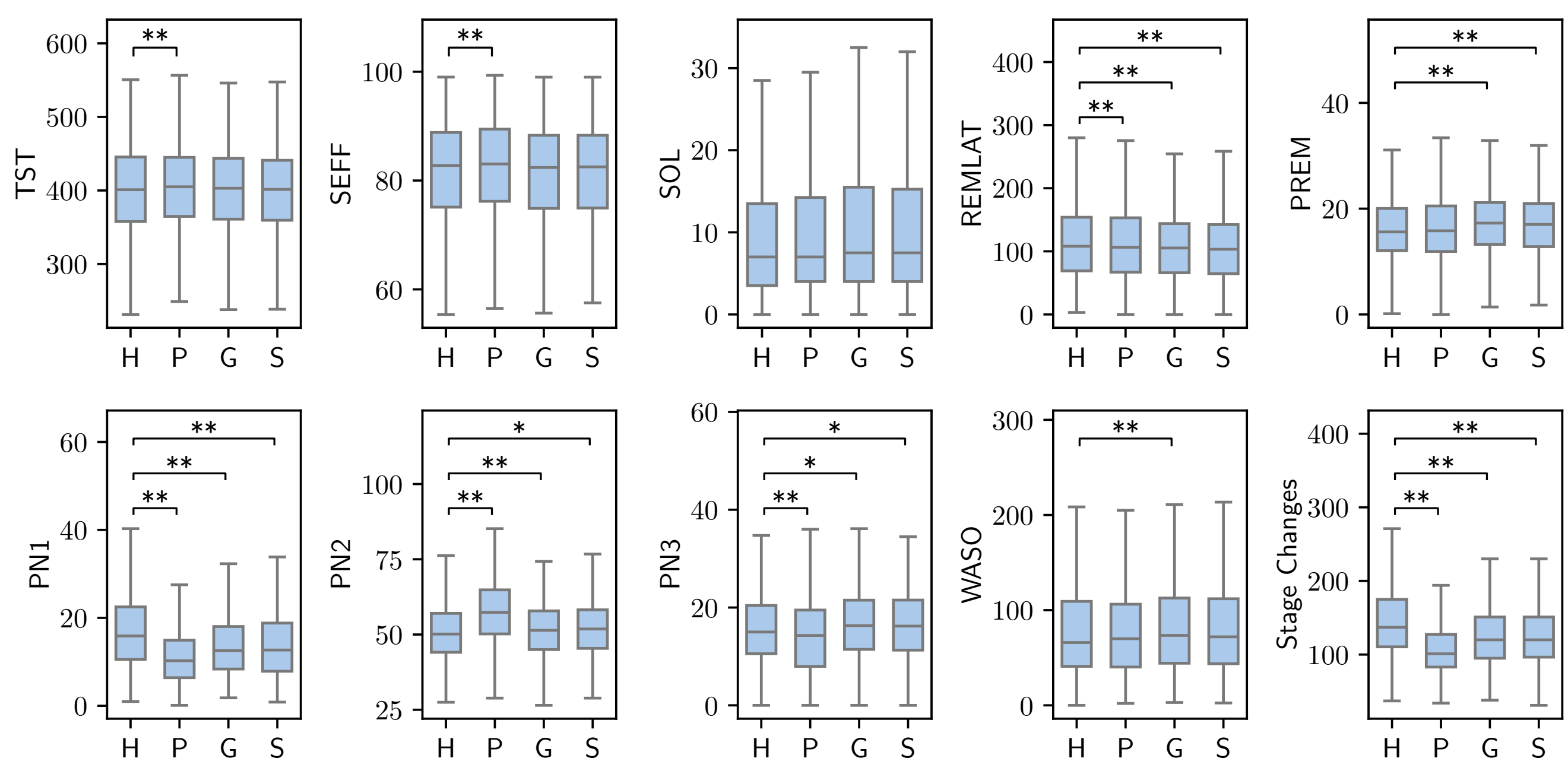

Figure S4 - *Sleep architecture measures per record for each model compared to human scorings. H = Human scorer, P = Pretrained Model, G = Generalized Model, S = Site Specific Model. Stars indicate a significant difference from the manual scorings. ** indicates a p-value < 0.01, while * indicates a p-value < 0.05.*

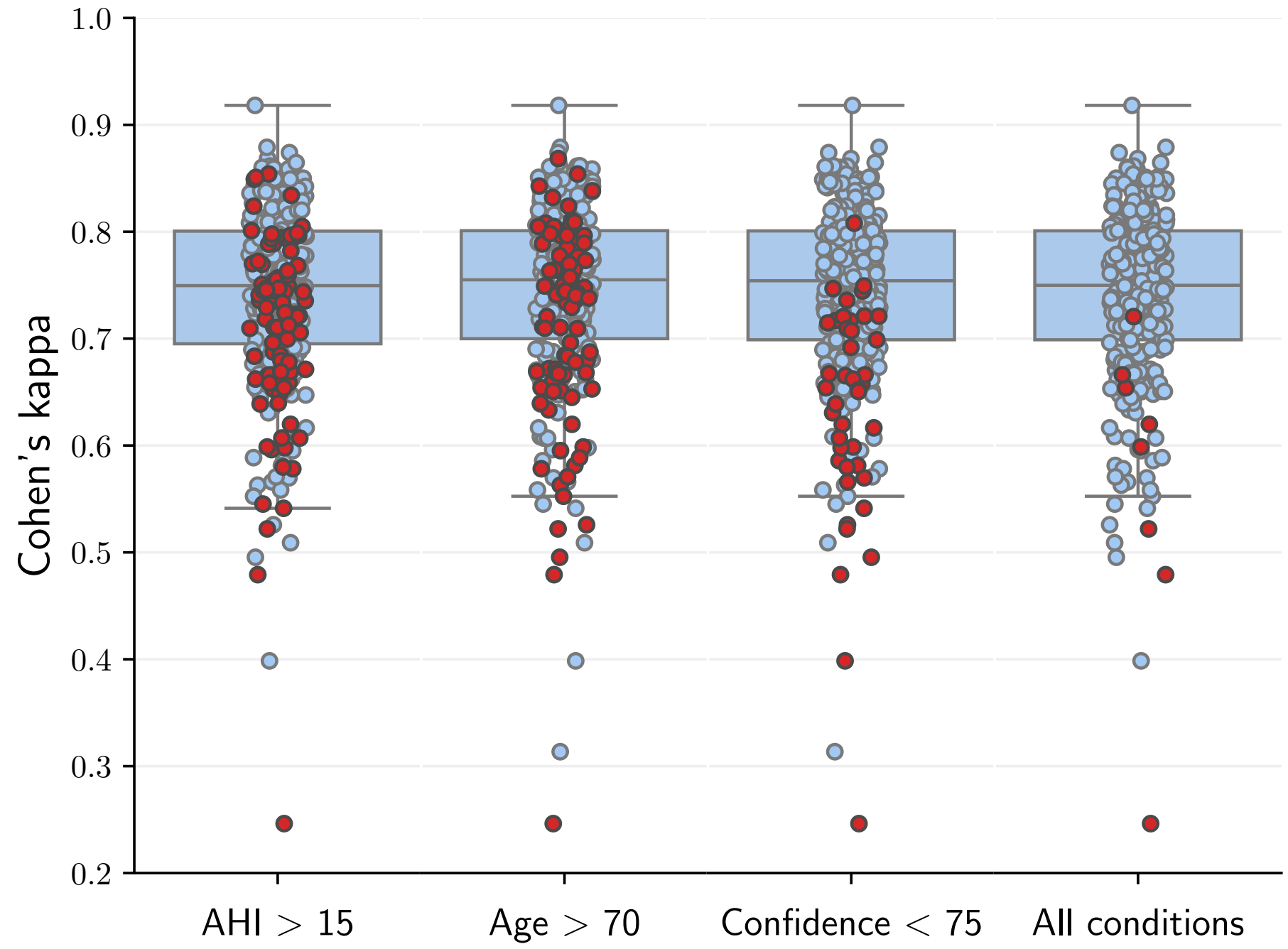

Figure S5 *- Distribution of mean subject-wise confidence scores for the significant variables included in the linear regression models (A, B and C). The red dots indicate individuals crossing the threshold for the particular variable. The recordings marked with red dots are considered at risk of being imprecisely staged by the model, as we observed linear relationships between Age and κ, AHI and κ, and per-night confidence and K.*

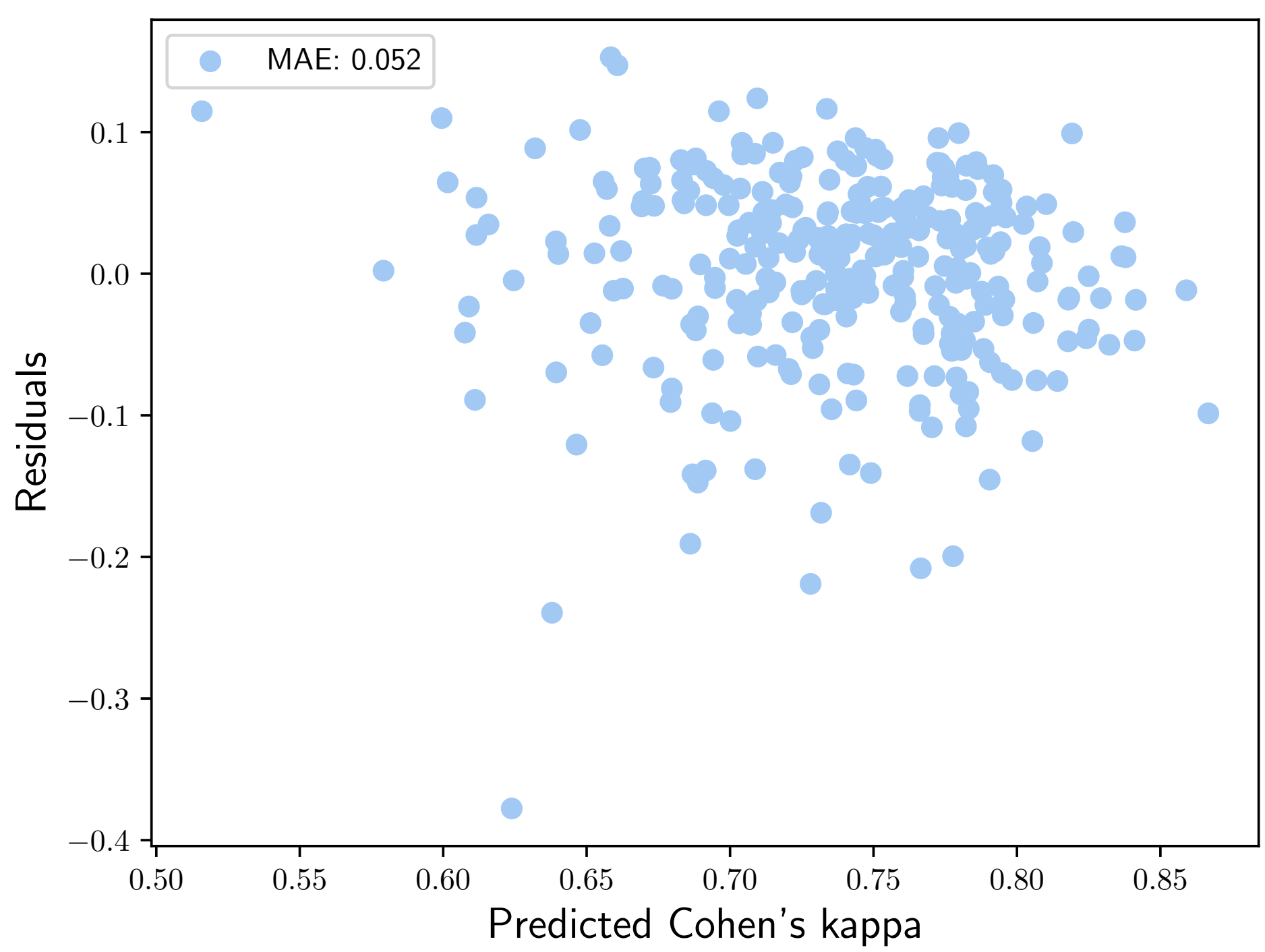

Figure S6 – *Residuals as a function of predicted Cohen's κ for model C, when fitting and testing on PACE and CBC with 10-fold cross-validation. The Mean Absolute Error (MAE) is 0.052, indicating that, on average, we can expect an error of 0.052 κ from the fitted model on new, unseen data.*

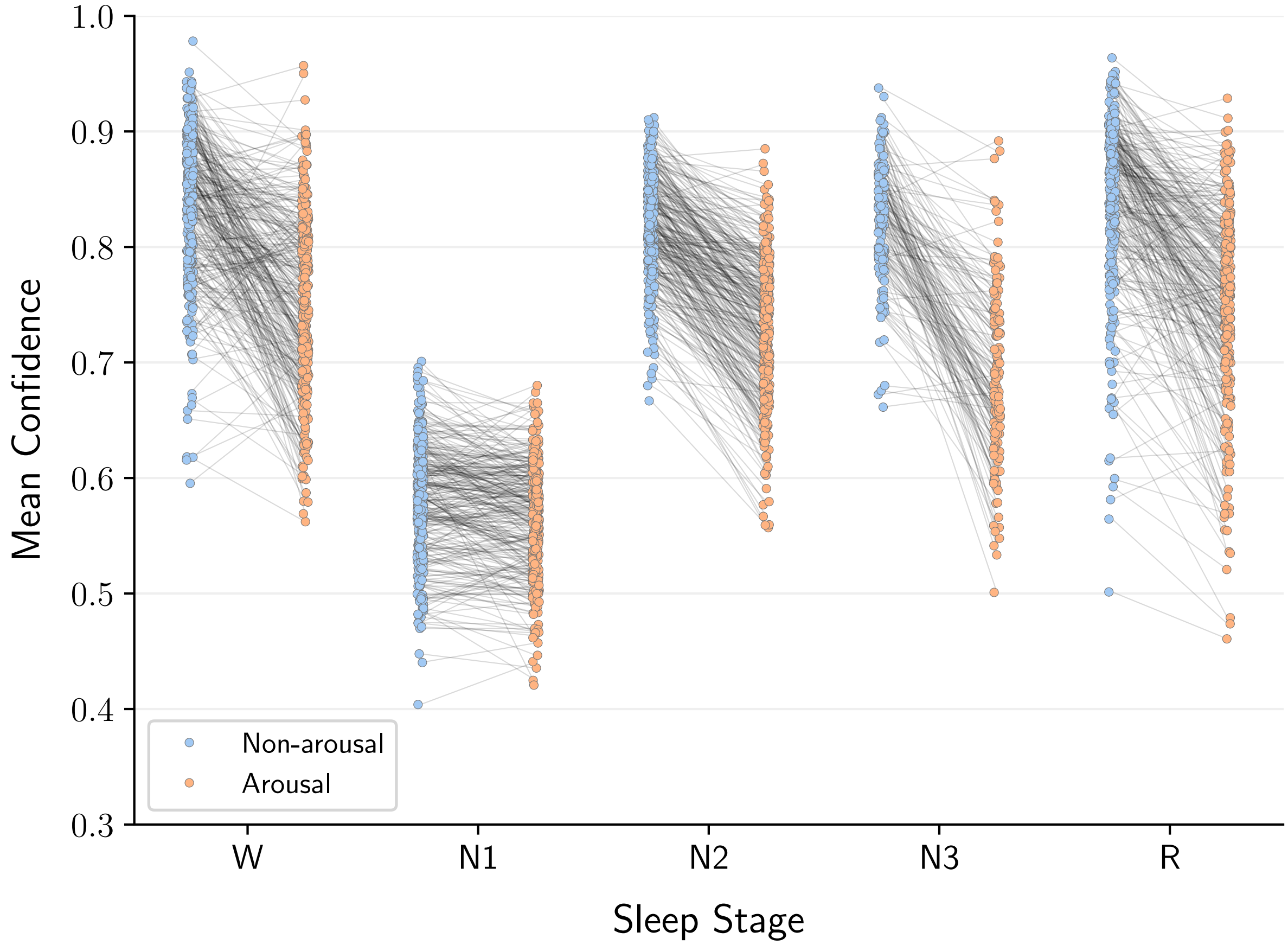

Figure S7 - *Model confidence across sleep stages for epochs affected by arousals compared to unaffected epochs. Epochs manually annotated as affected by arousals have significantly lower mean confidence across all sleep stages (p < 0.05). Each paired data point represents one stage from one PSG recording*

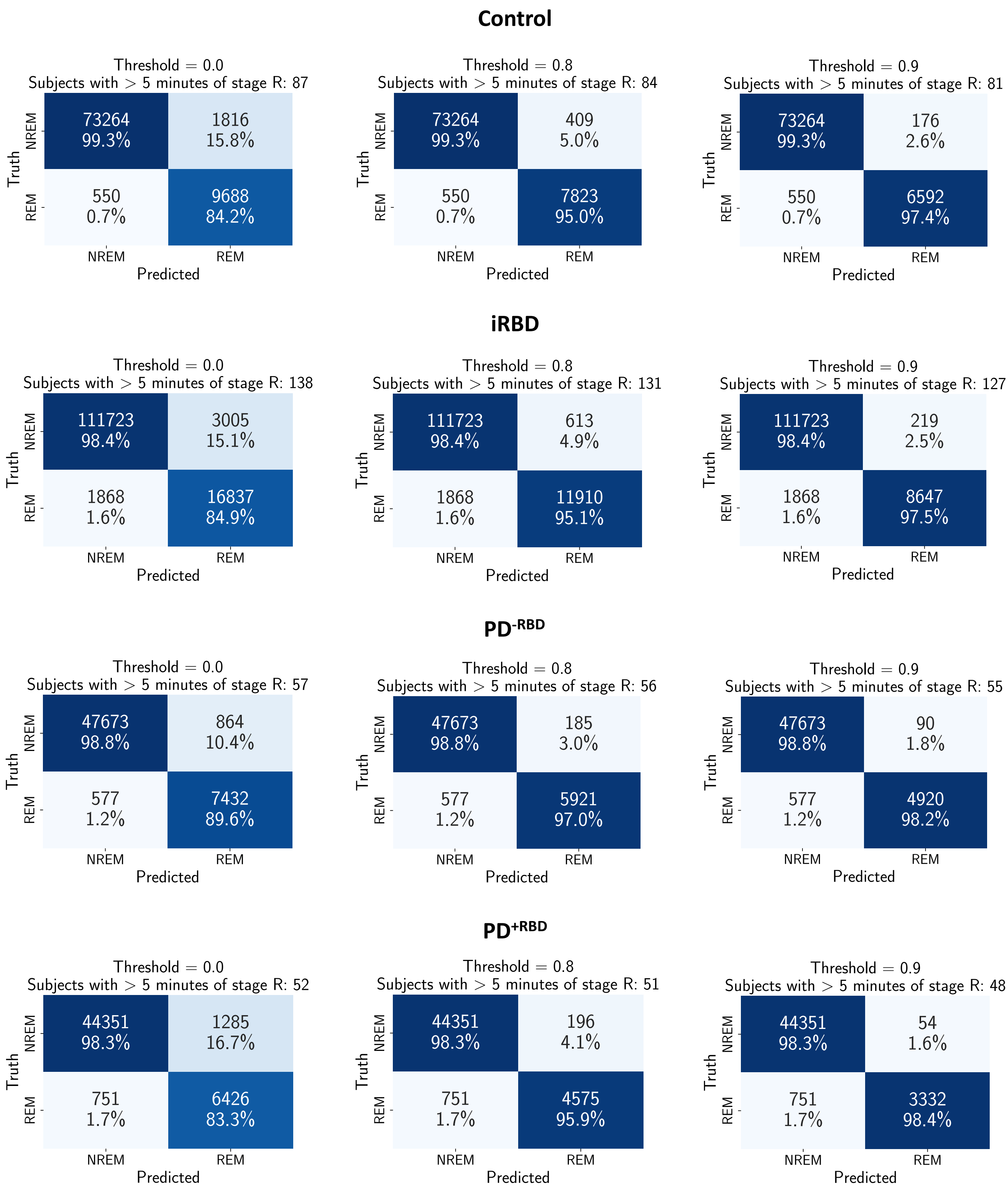

***Figure S8 –** Stage R confusion matrices for each clinical subgroup in the combined datasets of PACE and CBC, when subjected to three different confidence thresholds.*

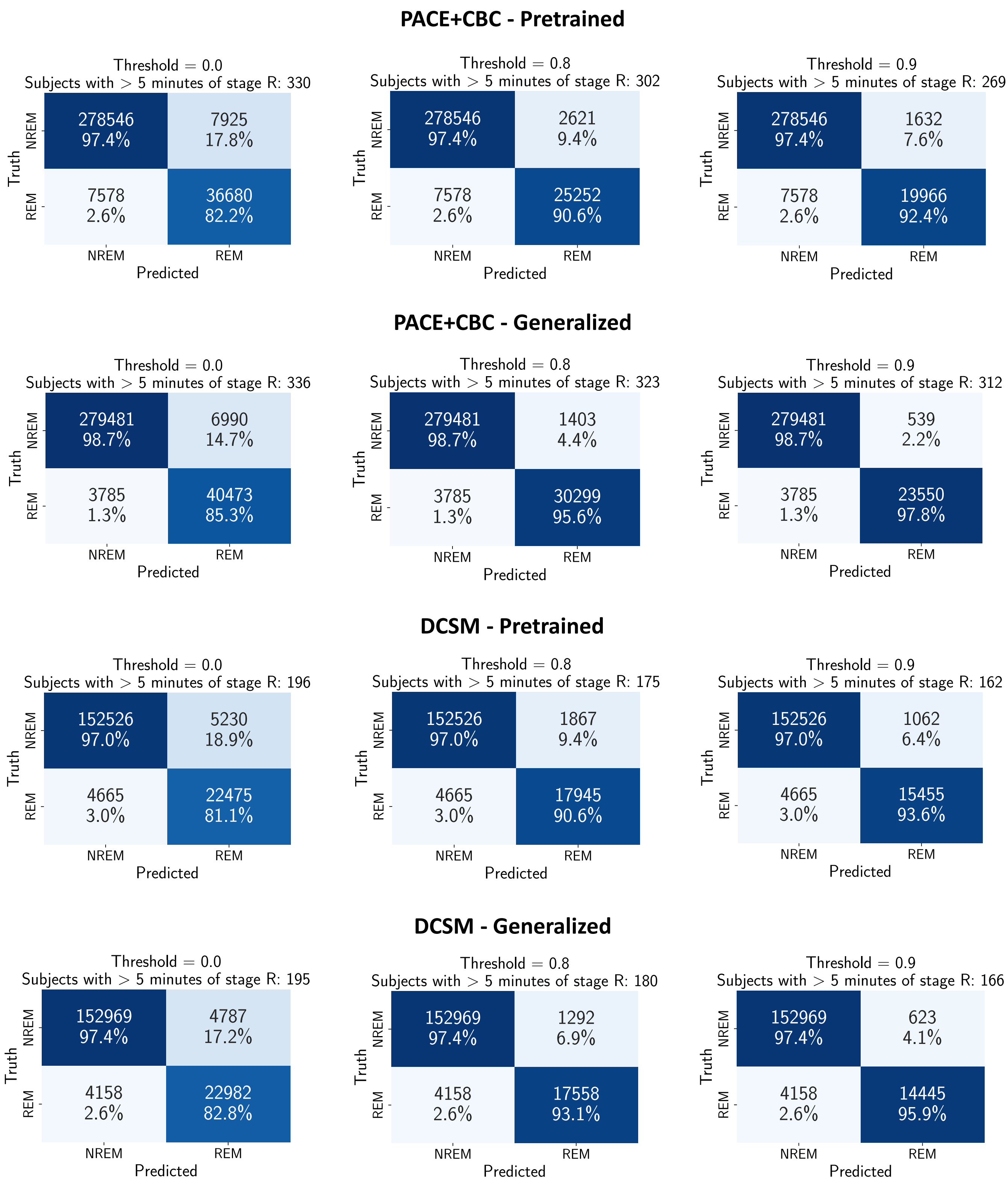

Figure S9 – Stage R confusion matrices for the PACE+CBC and DCSM datasets using the Pretrained and Generalized models at three different confidence thresholds. *Compared with the Pretrained Model, the Generalized Model achieved higher baseline REM sleep precision and larger gains in precision after thresholding while preserving ≥5 minutes of REM sleep in more subjects*
